# Precision sirolimus dosing in children: The potential for model-informed dosing and novel drug monitoring

**DOI:** 10.3389/fphar.2023.1126981

**Published:** 2023-03-20

**Authors:** Guofang Shen, Kao Tang Ying Moua, Kathryn Perkins, Deron Johnson, Arthur Li, Peter Curtin, Wei Gao, Jeannine S. McCune

**Affiliations:** ^1^ Department of Hematologic Malignancies Translational Sciences, City of Hope, and Department of Hematopoietic Cell Transplantation, City of Hope Medical Center, Duarte, CA, United States; ^2^ Alfred E. Mann School of Pharmacy and Pharmaceutical Sciences, University of Southern California, Los Angeles, CA, United States; ^3^ Clinical Informatics, City of Hope Medical Center, Duarte, CA, United States; ^4^ Division of Biostatistics, City of Hope, Duarte, CA, United States; ^5^ Division of Engineering and Applied Science, Andrew and Peggy Cherng Department of Medical Engineering, California Institute of Technology, Pasadena, CA, United States

**Keywords:** sirolimus (rapamycin), pediatrics, therapeutic drug monitoring (TDM), sweat, saliva, dried blood spots (DBS), pharmacogenomics, pharmacometabolomic

## Abstract

The mTOR inhibitor sirolimus is prescribed to treat children with varying diseases, ranging from vascular anomalies to sporadic lymphangioleiomyomatosis to transplantation (solid organ or hematopoietic cell). Precision dosing of sirolimus using therapeutic drug monitoring (TDM) of sirolimus concentrations in whole blood drawn at the trough (before the next dose) time-point is the current standard of care. For sirolimus, trough concentrations are only modestly correlated with the area under the curve, with *R* ^2^ values ranging from 0.52 to 0.84. Thus, it should not be surprising, even with the use of sirolimus TDM, that patients treated with sirolimus have variable pharmacokinetics, toxicity, and effectiveness. Model-informed precision dosing (MIPD) will be beneficial and should be implemented. The data do not suggest dried blood spots point-of-care sampling of sirolimus concentrations for precision dosing of sirolimus. Future research on precision dosing of sirolimus should focus on pharmacogenomic and pharmacometabolomic tools to predict sirolimus pharmacokinetics and wearables for point-of-care quantitation and MIPD.

## 1 Introduction

Rapamune^®^ (sirolimus) is approved to prevent organ rejection in patients aged 13 years or older receiving renal transplants by the Food and Drug Administration (Author Anonymous, 2022a). In addition, the European Medicines Agency (EMA) approved Rapamune^®^ for prophylaxis of organ rejection in adults at low to moderate immunological risk receiving a renal transplant and for treatment of patients with sporadic lymphangioleiomyomatosis with moderate lung disease or declining lung function (EMA, 2022). Over the past 20 years since these initial approvals, sirolimus has expanded to treat children undergoing heart (Rossano et al., 2017), hematopoietic cell (Monagel et al., 2021), intestine (Andres et al., 2021), liver (Hendrickson et al., 2019), or lung transplant (Hayes et al., 2014). Furthermore, children with vascular anomalies are treated with sirolimus (Mizuno et al., 2017c).

Sirolimus is a lipophilic macrocytic lactone that binds distinctly to FK binding protein 12 (FKBP12), forming a complex with the mammalian target of rapamycin (mTOR) (Cutler and Antin, 2004). This sirolimus–FKBP12–mTOR complex inhibits multiple cytokine–stimulated cell cycling pathways by reducing DNA transcription, DNA translation, protein synthesis, and cell signaling (Sehgal, 2003). It also inhibits interleukin–2–mediated proliferation signaling, leading to T–cell apoptosis (Sehgal, 2003).

The most common toxicities (>20%) in patients taking sirolimus include hypertriglyceridemia (45%–57%), stomatitis, diarrhea, abdominal pain, nausea, nasopharyngitis, acne, chest pain, peripheral edema, upper respiratory tract infection, headache, dizziness, and myalgia (Author Anonymous, 2022a). Sirolimus is available as tablets (0.5, 1, 2 mg) and, in some countries, as a liquid solution (1 mg/mL) formulation. The long half–life of sirolimus allows for convenient once–daily dosing, but a loading dose is required to rapidly achieve target drug concentrations in whole blood. Therefore, it is usually administered once daily at a fixed dose in adults (one 6–12 mg loading dose, followed by 2–4 mg daily) and as a body surface area–based dose in children (2.5 mg/m^2^/day) (Supplementary Table S1).

Sirolimus has a narrow therapeutic window. Its product labeling recommends monitoring sirolimus trough concentrations for all patients, especially those likely to have altered drug metabolism, in patients ≥13 years who weigh less than 40 kg, in patients with hepatic impairment, when a change in the sirolimus dosage form is made, and during concurrent administration of strong cytochrome P450 3A (CYP3A) inducers and inhibitors (Author Anonymous, 2022a). Therefore, its doses are personalized dosing using therapeutic drug monitoring (TDM) of whole blood obtained immediately before the subsequent dose (i.e., trough or predose samples). This provides a useful strategy to optimize transplant pharmacotherapy (Kahan et al., 2000). For the past 20 years, the majority of patients have undergone the following TDM process for precision dosing of sirolimus: 1. choose the target trough concentration in whole blood, typically between 3 and 14 ng/mL (Claxton et al., 2005; Alyea et al., 2008; Ho et al., 2009; Nakamura et al., 2012; Khaled et al., 2013); 2. administer a sirolimus loading dose based on weight or body surface area; 3. Obtain a trough pharmacokinetic sample; 4. quantitate the sirolimus concentrations in whole blood, typically using liquid chromatography–mass spectrometry (LC–MS); 5. use that trough concentration to personalize the dose to achieve the target trough concentration. Notably, sirolimus whole blood concentrations may be measured by either chromatographic or immunoassay methods (Mahalati and Kahan, 2001; Schmid et al., 2009). Due to cross–reactivity with sirolimus metabolites, immunoassay methods have a positive bias ranging from 14% to 39% compared to LC–MS methods (Schmid et al., 2009). Because sirolimus whole blood concentrations vary by the type of assay used, concentrations are not interchangeable between methods. Therefore, sirolimus TDM should be conducted using one consistent bioanalytical method within an institution.

## 2 Why should we expand the precision dosing of sirolimus beyond TDM

Dosing sirolimus based on trough concentrations has been the current standard of care (Stenton et al., 2005) for over 20 years. However, trough concentrations only modestly correlate with AUC_0–24hr_, with *R* ^2^ values between trough concentrations and area under the plasma concentration-time curve for sirolimus ranging from 0.52 to 0.84 (Schachter et al., 2004; Schubert et al., 2004; Goyal et al., 2013). We have not found publications regarding the existence or results from a sirolimus proficiency program that evaluates the accuracy of quantitation, pharmacokinetic modeling, and dose recommendations. Hopefully, such a proficiency program will be developed for sirolimus TDM because such programs have discovered challenges with TDM of other drugs (Neef et al., 2006; McCune et al., 2021b).

Although TDM is accepted for sirolimus, trough concentrations are limited because they fail to provide a rich, mechanistic description of the pharmacokinetic/pharmacodynamic relationship (Dupuis et al., 2013) that could advance our understanding of why certain patients experience adverse outcomes. Model-informed precision dosing (MIPD) may improve clinical outcomes by optimizing the personalized dose for an individual patient (Darwich et al., 2017). The development of such mechanistic models can help improve individual patients’ clinical outcomes, which can be attributed to the pharmacokinetics and pharmacodynamics of sirolimus. Regarding the pharmacokinetics, low immunosuppressant concentrations or exposure is multifactorial; they can result from insufficient dosing or dose personalization, aberrant pharmacokinetics due to patient covariates, and/or non-adherence (McCune and Bemer, 2016; McCune et al., 2016; Vaisbourd et al., 2022). Non-adherence is associated with the development and severity of adverse outcomes such as graft loss in solid organ transplant (Foster et al., 2018) and graft-versus-host disease (GVHD) hematopoietic cell transplant patients (Gresch et al., 2017; Ice et al., 2020). Thus, improvements in sirolimus dosing to minimize the between-patient variability in sirolimus concentrations through novel -omics techniques or point-of-care monitoring at home may be beneficial. These will be the focus of this review.

However, the promising innovations in -omics techniques and mathematical modeling related to the immune system necessitate that we briefly describe factors possibly affecting the pharmacodynamics of sirolimus. Pharmacodynamic monitoring of the cellular targets of immunosuppressant drugs may reflect clinical outcomes better than TDM (Monchaud and Marquet, 2009a; Monchaud and Marquet, 2009b). For example, recipient pretransplant inosine monophosphate dehydrogenase activity is associated with clinical outcomes after renal transplant or allogeneic hematopoietic cell transplant recipients treated with mycophenolate mofetil (MMF) (Glander et al., 2004; Bemer et al., 2014). However, a drug-specific pharmacodynamic biomarker for sirolimus’s effectiveness or toxicity has yet to be identified. Turning to the use of sirolimus to alter the immune system, the ontogeny of innate and adaptive immune responses involve more than 1,600 genes (Simon et al., 2015). These genes are essential to sustain life in a hostile environment. Yet the immune system is relatively immature at birth. It has to evolve during a lifetime of exposure to many foreign challenges through childhood, young and mature adulthood (including pregnancy). It subsequently declines in old age (Simon et al., 2015). Beyond the effects of these genes, patients may have several transcriptomic (Furlan et al., 2020), proteomic (Cohen Freue et al., 2013; Levitsky et al., 2013), metabolomic [reviewed in Supplementary Table S1 of McCune et al. (2021a)], and lipidomic (Liggett et al., 2022) characteristics that could influence the effectiveness and toxicity of transplantation and/or sirolimus. For example, metabolomics can offer discoveries yielding new insights into how metabolites (here, endogenous, not sirolimus metabolites) influence gut physiology, organ function, and immune function (Wishart, 2019). Given their signaling properties in addition to a multitude of other functions (Cockcroft, 2021), lipidomics may enable a deeper mechanistic understanding of the T-cell migration (Cucchi et al., 2020), drug-target interactions, and systems physiology from the molecular (genomic, proteomic, metabolomic) to cellular to whole-body levels. Collectively, these works could lead to a system-wide perspective of pathophysiology wherein genes, proteins, metabolites, and lipids are understood to interact synergistically to modify the functions within a patient receiving sirolimus. These insights may provide the foundation for enhanced pharmacokinetic/dynamic modeling to comprehensive quantitative systems pharmacology (QSP) models (Ayyar and Jusko, 2020). In homogenous and sufficiently powered populations of patients treated with sirolimus, multi-omic tools should be explored for precision dosing and for building QSP models to improve clinical outcomes.

Mathematical modeling and simulation can characterize the complexity and multiscale nature of the mammalian immune response and provide a mechanistic understanding of the data generated from the novel–omics technologies (Palsson et al., 2013). As an example of such modeling and simulation, the Fully-integrated Immune Response Model (FIRM) represents a multi-organ structure comprised of the target organ, where the immune response occurs, and circulating blood, lymphoid T, and lymphoid B tissue (Palsson et al., 2013). FIRM can be expanded to include novel biological findings relevant to sirolimus, such as incorporating novel medications that target antigen-presenting cells (B-cells), T-cell subsets, T-cell signal transduction, costimulatory molecules, or cytokines into QSP models. Early steps are being taken to apply QSP models to precision dosing, specifically in the context of the well-characterized coagulation cascade (Hartmann et al., 2016). However, the inherent complexity of the immune system and the difficulty of measuring many aspects of a patient’s immune state *in vivo* makes it challenging to develop such QSP models of immune response (Laubenbacher et al., 2022). Because the immune system has an important role in such a wide range of diseases and health conditions, digital twins of the immune system are of keen interest. Advanced medical digital twins will make precision medicine a reality (Laubenbacher et al., 2022).

Therefore, it is crucial to explore newer methods for precision dosing of sirolimus (Table 1). Here, we summarize our findings from a series of literature reviews (Supplementary Methods and Supplementary Figures S1–S5) about various –omic or point–of–care tools focusing on the sirolimus pharmacokinetics that may improve the precision dosing of sirolimus in children.

**TABLE 1:** Steps of and research about precision sirolimus dosing: TDM of sirolimus dosing the initial (first) dose to achieve the target trough. The tools in the bold font should be implemented, and those in the italicized font are not recommended for clinical use.

Steps	Current steps	Research opportunities
1. Choose and then administer the initial sirolimus dose	Body weight	Before sirolimus administration:
		**Model-informed precision dosing using population pharmacokinetic (popPK)** Djebli et al. (2006), Mizuno et al. (2017a), and Darwich et al. (2017)
		Pharmacogenomics: Table 2
		Pharmacometabolomics
2. Pharmacokinetic blood sampling	Trough	PopPK–guided limited sampling schedules for blood sampling Djebli et al. (2006)
		*Dried blood spots*: Table 3
		Saliva sampling: Table 4
		Sweat sampling: Table 5
3. Quantitation of sirolimus concentrations	Immunoassay	Metabolite–specific antibody–like molecularly imprinted polymers and redox–active reporter nanoparticles Wang et al. (2022)
	LC-MS	
4. Pharmacokinetic modeling of concentration–time data	Not possible with trough concentration only	**PopPK–guided dosing with a posterior Bayesian prediction** Mizuno et al. (2017a)
5. Determine one patient’s precision dose to achieve their target trough	$Personalized\;Dose=Target\;trough\;X\;\left(\frac{Initial\;Dose}{Trough\;with\;initial\;dose}\right)$	

## 3 Sirolimus pharmacokinetics

After oral administration, sirolimus achieves its peak whole blood concentrations within 1–3.5 h (MacDonald et al., 2000; Stenton et al., 2005). The apparent oral bioavailability of sirolimus is poor (Stenton et al., 2005). Extensive intestinal and hepatic first-pass metabolism contributes to the low oral bioavailability.

Sirolimus is highly lipophilic (log P of 4.3, Supplementary Table S1). Sirolimus has a large apparent volume of distribution of (5.6–22.8 L/kg) (Brattstrom et al., 2000; Stenton et al., 2005). This large distribution volume implies extensive distribution to organs and tissues and contributes to the long half-life. The free fraction in plasma is 8% (MacDonald et al., 2000). Sirolimus is partitioned extensively into blood cells with a blood-to-plasma ratio of 35.6 (Tejani et al., 2004). In kidney transplant (KT) patients, this ratio is independent of sirolimus concentration and exhibits a large variability (Ferron et al., 1997). In humans, sirolimus is distributed among red blood cells (94.5%), whole blood (3.1%), lymphocytes (1.01%), and granulocytes (1.0%) (Stenton et al., 2005). The sequestration of sirolimus in red blood cells is believed to be partially due to their rich content of immunophilins (Stenton et al., 2005). In the whole blood compartment, sirolimus exhibits concentration–dependent binding to lipoproteins (40%) with a minor fraction (<4%) bound to plasma proteins. Therefore, whole blood is considered the most favorable matrix for TDM (Stenton et al., 2005).

The primary route of elimination occurs *via* fecal/biliary pathways, with an estimated terminal elimination half–life of approximately 62 ± 16 h (Stenton et al., 2005). After a single oral dose in healthy adults, sirolimus has a half-life of 81.5 h and a total body clearance is 278 mL/h/kg (Brattstrom et al., 2000).

Sirolimus is metabolized by CYP3A4 and CYP3A5 in both human liver and small intestinal microsomes to various demethylated and hydroxylated species (Paine et al., 2002). Degradation products, including an ester hydrolysis product and a ring–opened isomer, have also been described (Paine et al., 2002). In the liver, sirolimus is primarily metabolized by CYP3A4, with CYP3A5 and CYP2C8 having lesser roles (Jacobsen et al., 2001; Emoto et al., 2013). Sirolimus is transported by the multidrug resistance gene product pump p–glycoprotein (PgP) (Stenton et al., 2005), an apically directed ATP–dependent transmembrane secretory (efflux) pump expressed at high levels in enterocytes (Paine et al., 2002).

Examples of potential drug–drug interaction (DDI) with sirolimus result from concomitant calcium channel blockers, imatinib, antibiotics, or antifungals (Leather, 2004; Bernard et al., 2014; Bleyzac et al., 2014). Because of routine TDM of trough concentrations of the sirolimus, these results can be used to identify a DDI and appropriately personalize the sirolimus dose. For example, the DDI between azole antifungals and sirolimus has long been recognized (Yee and McGuire, 1990b; Yee and McGuire, 1990a) and can be managed through TDM (Leather, 2004). The azoles have variable CYP3A4 inhibition, potentially affecting CYP2C9, CYP2C19, and PgP (Leather, 2004). Although these azole–immunosuppression DDI are well known, their management can be variable and could benefit from improved pharmacokinetic/pharmacodynamic modeling. The EMA changed the drug label for sirolimus to include therapeutic monitoring during dose adjustments when inducers or inhibitors of CYP3A are concurrently administered and/or discontinued (Ehmann et al., 2014).

Furthermore, sirolimus is also susceptible to being the victim drug to natural products altering CYP3A or PgP activity, such as grapefruit juice and St. John’s wort (Edwards et al., 1999; Mai et al., 2003; Brantley et al., 2013). Sirolimus pharmacokinetics may have circadian variability, as the maximum plasma concentration and AUC of other CYP3A/PgP substrates (i.e., cyclosporine and tacrolimus) are higher in the morning than in the afternoon (Baraldo and Furlanut, 2006). Seasonal variation is also of concern, as it has recently been reported that duodenal CYP3A4 mRNA is significantly higher between April and September than between October and March (Thirumaran et al., 2012).

The maintenance dose of sirolimus should be adjusted in patients with hepatic impairment or at risk of interactions with sirolimus, either due to concomitant drugs (Author Anonymous, 2022a) or natural products (Paine et al., 2018) affecting CYP3A or PgP activity.

## 4 Model-informed precision dosing of sirolimus in children

Pharmacometrics enables developing models that describe factors affecting the pharmacokinetics and/or pharmacodynamics in children (Mehrotra et al., 2016). Children are not small adults because of differences in biochemical, body composition, and physiology processes (Rodman et al., 1993; Murry et al., 1995; de Wildt et al., 1999; Blanco et al., 2000; Kearns et al., 2003; Ince et al., 2009; Mahmood, 2014). Specific to sirolimus, the data regarding the maturation of CYP3A4 is conflicting, with different age variations in enzyme activity. For example, Salem et al. (2014) suggested that hepatic CYP3A4 increases from an early age and reaches the adult level by 2.5 years. In contrast, Upreti and Wahlstrom (2016) suggested that CYP3A4 maturation exceeds the adult level between 0.1 and 11 years. Using such enzyme maturation data is critical in creating physiologically based pharmacokinetic models (PBPK) to predict pediatric pharmacokinetics and dosing (Mehrotra et al., 2016).

A PBPK model for children aged 1 month to 2 years demonstrated that the relationship between allometrically scaled *in vivo* sirolimus clearance and age was described by the Emax model (Emoto et al., 2015a). Consistent with this increased clearance in patients, *in vitro* intrinsic clearance of sirolimus using pediatric liver microsomes shows a similar age-dependent increase. In children older than 2 years, allometrically scaled apparent oral clearance of sirolimus did not show further maturation. Simulated clearance estimates with a sirolimus PBPK model that included CYP3A4/5/7 and CYP2C8 maturation profiles were in close agreement with observed *in vivo* clearance values (Emoto et al., 2015a). However, further exploration of the impact of different assumptions regarding CYP3A4 age–related changes on the PBPK model predictive performance may be beneficial (Lang et al., 2021). In addition, PBPK model-simulated sirolimus pharmacokinetic profiles predicted the actual observations well (Emoto et al., 2015a). The mean sirolimus clearance was 11 ± 3 L/h, 17 ± 4 L/h, 21 ± 3 L/h, and 18 ± 6 L/h for the age groups of 1–8 months (<1), 1 year (1 to <2), 2 years (2 to <3), and 3–18 years (≥3), respectively (Emoto et al., 2015a). Sex, ethnicity, or race did not show a statistically significant association with sirolimus clearance in a cohort of 44 children (Emoto et al., 2015a). These results demonstrate the utility of a PBPK modeling approach for predicting the developmental trajectory of sirolimus metabolic activity and its effects on total body clearance in neonates and infants (Emoto et al., 2015a).

Complementing PBPK modeling, population pharmacokinetic (PopPK) models (Beal and Sheiner, 1982) can address relevant hurdles by accounting for variability and mitigating the resource intensity obtaining more pharmacokinetic samples beyond trough samples. PopPK models mathematically describe typical drug kinetics while accounting for between subject variability and residual unknown variability (Holford et al., 2000) and the role of demographic covariates responsible for or related to variability, such as age or gender. PopPK models also facilitate the development of limited sampling schedules, which is essential since most immunosuppression is administered in the outpatient clinic (Li et al., 2012; Li et al., 2013). PopPK models could overcome the major challenge of the –omics tools, specifically the interference from confounding factors (Ioannidis et al., 2009; Gu et al., 2012). Pharmacokinetics can be used to address these confounding factors by identifying factors associated with aberrant metabolism. Theoretical allometrically scaled body weight accounted for differences in body size (Mizuno et al., 2017b). Using a popPK model and more comprehensive blood sampling of sirolimus, MIPD was conducted in children with vascular anomalies as part of a prospective phase II trial (Mizuno et al., 2017a). In 52 children, the target trough was attained in 94%, specifically 49 of 52 children, across the age range of 3 weeks–18 years after 2–3 months of therapy (Mizuno et al., 2017a). The mean sirolimus dose to achieve the target trough of ∼10 ng/mL for patients older than 2 years was 1.8 mg/m^2^ twice daily (range 0.8–2.9), while it was 0.7–1.6 mg/m^2^ twice daily for patients 3 weeks of age to 2 years. The final popPK model included a maturation function for sirolimus clearance and allometrically scaled body weight to account for size differences. The mean allometrically scaled sirolimus clearance estimates increased from 3.9 to 17.0 L/h/70 kg with age from shortly after birth to 2 years of age, while the mean estimate for patients older than 2 years was 18.5 L/h/70 kg. This MIPD can be extended to other pediatric populations and perhaps adults (Mizuno et al., 2017a).

## 5 Multi-omics technologies

### 5.1 Pharmacogenomics

The pharmacokinetics and pharmacodynamics of sirolimus can be influenced by genetic polymorphisms influencing the expression of CYP3A4, CYP3A5, or PgP (Utecht et al., 2006). Pre–emptive genotyping, or using genotyping results to guide initial dosing before sirolimus administration, takes a step towards the “right–dose–first–time” paradigm (Minto and Schnider, 1998). However, preemptive genotype–directed dosing will not account for non-genetic factors associated with sirolimus pharmacokinetics (Section 3 and Section 4). Although the *CYP3A4* and *MDR1* genes may contribute to sirolimus pharmacokinetics, we focused on *CYP3A5* because it is the only gene involved in sirolimus pharmacokinetics with Clinical Pharmacogenetics Implementation Consortium (CPIC^®^) guidelines. We reviewed the literature regarding the association of the *CYP3A5* genotype with the pharmacokinetic phenotype (Supplementary Figure S1). Very few studies evaluated if the *CYP3A5* genotype was associated with the effectiveness or toxicity (i.e., pharmacodynamics) of sirolimus–based immunosuppressive regimens (Mourad et al., 2005; Renders et al., 2007; Lukas et al., 2010; Zochowska et al., 2012; Wang et al., 2014; Khaled et al., 2016; Rodriguez-Jimenez et al., 2017).

A variant in intron 3 of *CYP3A5* (rs776746) creates a cryptic splice site which results in aberrant splicing and creates a premature stop codon that results in transcript degradation (Hustert et al., 2001; Kuehl et al., 2001). This allele, now known as CYP3A5*3, explains the liver’s highly variable expression of CYP3A5 protein. Based on homozygosity for the CYP3A5*3 allele, individuals are divided into CYP3A5 non-expressors (CYP3A5*3/*3) and CYP3A5 expressors (CYP3A5*1/*3 and CYP3A5*1/*1) (Rodriguez-Antona et al., 2022). CYP3A5*3 is the most common allele in European and Asian populations, but it is the minor allele in people of African ancestry. Thus, CYP3A5 protein is only expressed in 10%–30% of Europeans and Asians but in ∼70% of people of African ancestry (Rodriguez-Antona et al., 2022).

The *CYP3A5* rs776746 variant has been the most extensively evaluated for its association with sirolimus pharmacokinetics in transplant patients (Table 2). CYP3A5 non-expressors should have lower sirolimus clearance and, without TDM, should have higher dose–adjusted trough concentrations (Anglicheau et al., 2005). Conversely, the CYP3A5 expressors should have CYP3A5 protein and thus faster sirolimus clearance, and, without TDM, should have lower dose–adjusted trough concentrations. Anglicheau (Anglicheau et al., 2005) was one of the earliest and largest (*n* = 129) studies to demonstrate significant differences in dose–adjusted trough concentrations between the *CYP3A5* genotypes. They evaluated three different KT patient treatment groups: 1) sirolimus rescue therapy after discontinuing calcineurin inhibitor (CNI) therapy for concerns of nephrotoxicity (*n* = 69), 2) sirolimus-based therapy *de novo* post–KT (*n* = 51), 3) sirolimus + CNI–based therapy (*n* = 29). In the rescue therapy group, expressors had an average weight adjusted trough concentration of 89 ± 65 (ng/mL)/(mg/kg) vs. non-expressors 145 ± 93 (ng/mL)/(mg/kg) (*n* = 69, *p* < 0.02) at 3 months post sirolimus initiation. Notably, only those patients who were transitioned onto sirolimus rescue therapy had a statistically significant association between *CYP3A5* rs776746 genotype and dose–adjusted trough concentration (Anglicheau et al., 2005).

**TABLE 2:** Pharmacogenetic association of *CYP3A5* rs776746 genotype with sirolimus pharmacokinetics. Publications are organized in order of publication date, starting with the oldest. Gray-shaded cells show results where the *CYP3A5* genotype was associated with a statistically significant difference (*p* < 0.05) in sirolimus pharmacokinetics.

**Author**	**Study design/** **Study population**	**Sirolimus dosing and pharmacokinetic sampling**	**Immunosuppressant regimen^a^**	***CYP3A5* Allele**	**Sirolimus PK endpoint**	
Anglicheau et al. (2005)	Retrospective N= 149 KT Self–reported race: 140 Caucasian, 4 Black, 5 Caribbean Age: 44.9 ± 11.4 years Child: No Included: SIR for ≥3 months Excluded: (*n* = 8) DDI (i.e., nicardipine, diltiazem, fluconazole) A priori power analysis: NA	Initial dose: NA Target trough: SIR Based (including Rescue Therapy): 10–20 ng/mL *SIR + CNI–Based*: 10–15 ng/mL PK sampling: Whole blood collected 24 hours post-dose; used troughs after at least months of SIR administration Was PK sampling at steady state?: Yes Quantitation: HPLC Detection: NA LOD/LOQ: NA			**Trough/(Dose/Weight)** (ng/mL) per (mg/kg) Mean ± SD	
			SIR-Based Therapy: N=51^b^ SIR, purine inhibitor (azathioprine or MMF), + Prednisolone Steroid dosing^a^: NA	*1/*1 *1/*3 *n* = 13^b^	147 ± 55	
				*3/*3 *n* = 18^b^	171 ± 113	
			*SIR Rescue Therapy* N = 69 SIR is used in pts with suspected CNI nephrotoxicity; Steroid dosing: NA	*1/*1 *1/*3 *n* = 11	89 ± 65	
				*3/*3 *n* = 58	145 ± 93	
			*SIR + CNI–Based Therapy*: N=9: SIR + TAC + Prednisolone N = 20: SIR + Cyclosporine + Prednisolone Steroid dosing: NA	*1/*1 *1/*3 *n* = 7	264 ± 221	
				*3/*3 *n* = 22	268 ± 141	
Mourad et al. (2005)	Cross–Sectional Study N = 85 KT Self–reported race: 82 Caucasian, 2 African, 1 South Asian Age: 52.3 ± 13 years Child: No Included: stable post–KT, 6.2 – 285.3 months post–KT Excluded: History of graft rejection or altered renal function leading to modifying drug doses during 2 months before the study; pts taking drugs that precipitate DDI A priori power analysis: NA	Initial dose: NA Target trough: 5–15 ng/mL PK sampling: whole blood collected 12-hour post–dose Was PK sampling at steady state?: NA Quantitation: LC–MS/MS LOD/LOQ: NA	ISx regimen: SIR with Prednisolone (*n* = 81), and MMF (*n* = 27) or Azathioprine (*n* = 12); SIR and tacrolimus (*n* = 24)^c^ Steroid dosing: Weight–adjusted prednisolone dose per day was not significantly different between the *CYP3A5* expressors vs. non–expressors	**Allele**	**Trough/(Dose/Weight)** (ng/mL) per (mg/kg) Median (range)	
				*1/*1 *1/*3 *n* = 7^c^	176 (102 – 260)	
				*3/*3 *n* = 78^c^	169 (46.2 – 1093)	
Le Meur et al. (2006)	Clinical Trial N= 21 KT Self–reported race: 21 Caucasian Age: *3/*3: 51.0 ± 13 years *1/*1 &*1/*3: 40.5 ± 17.3 years Child: No Inclusion: NA Excluded: pts taking drugs that precipitate DDI A priori power analysis: NA	Initial dose: 15 mg/day loading dose days 1 and 2, 10 mg/day ×7 days, then titrated to target trough Target trough: 10–15 ng/mL PK sampling: Whole blood was collected immediately before dose administration, then at 0.33, 0.66, 1, 1.5, 2, 3, 4, 6, 9 hours after dose administration, on weeks 1 (W1), week 2 (W2), 1 month 1 (M1), 3 months (M3). In W1 and W2, two additional samples were obtained at 12 and 24 hours. Was PK sampling at steady state?: NA Quantitation: LC–MS LOD: 0.5 ng/mL LOQ: 1 ng/mL	ISx regimen: SIR, MMF, Thymoglobulin × 5 days, steroids Steroid dosing: Methylprednisolone 250 mg IV pre– and post–surgery, then oral prednisolone 1 mg/kg/day days 1–7, 0.5 mg/kg/day days 8–14, taper by 5 mg/day each week down to 20 mg/day, decrease by 2.5 mg/day each week down to 10 mg/day, dose maintained for 1 month then decrease by 2.5 mg/day each week until complete stop if possible.	**Allele**	**Trough/Dose** (ng/mL) per (mg) Mean (range)	
				*1/*1 *1/*3 *n* = 3	W1: 0.45 (0.35–1.23) W2: 0.34 (0.13–0.37) M1: 0.85 (0.50–0.87) M3: 0.94 (0.33–1.30)	
				*3/*3 *n* = 18	W1: 1.53 (0.78–5.44) W2: 1.61 (0.50–9.10) M1: 2.16 (1.06–5.07) M3: 2.56 (0.92–6.66)	
Renders et al. (2007)	Prospective clinical study N= 20 KT Self–reported race: 20 Caucasian Age: 54.1 ± 11.1 years Child: No Included: clinically stable pts, who had reached steady-state on SIR Excluded: NA A priori power analysis: NA	Initial dose: NA Target trough: NA PK sampling: trough (n=20); AUC (n=10 of the original 20 pts): before and 0.5, 1, 2, 3, 4, 6, 8, 9, 10, 11, 12, and 24 hours after administration of a single dose of sirolimus Was PK sampling at steady state?: Yes Quantitation: LC/MS LOD/LOQ: NA	ISx regimen: SIR ± MMF ± Prednisolone Steroid dosing: Prednisolone: 5–10 mg/day	**Allele**	**Dose/Trough** (× 10^3^ L) Mean ± SD	
				*1/*1 *n* = 0^d^	0.6 ± 0.1	
				*1/*3 *n* = 4	0.4 ± 0.2	
				*3/*3 *n* = 16	0.6 ± 0.4	
					**AUC**_**0–24hr**_ **/Dose** (ng × hr /mL per mg)	
				*1/*1 *1/*3 *n* = 3	56.3 ± 5.3	
				*3/*3 *n* = 7	118 ± 81.8	
Miao et al. (2008)	Clinical Trial N= 47 KT Self–reported race: 47 Chinese (Han nationality) Age: 42 ± 15 years Child: No Included: KT pts, stable graft function Excluded: pts taking drugs that precipitate DDI with SIR, except 3 pts receiving CNI + SIR A priori power analysis: NA	Initial dose: NA Target trough: NA PK sampling: NA Was PK sampling at steady state?: NA Quantitation: HPLC LOD/LOQ: NA	ISx regimen: SIR, MMF, steroids Steroid dosing: NA	**Allele**	**Trough/(Dose/Weight)** (ng/mL) per (mg/kg) Mean ± SD	
				*1/*1 *1/*3 *n* = 21	318 ± 113	
				*3/*3 *n* = 26	397 ± 129	
Zochowska et al. (2012)	Retrospective N = 100 KT Self–reported race: NA (Poland) Age: 48.4 ± 11.5 years Child: No Included: KT Excluded: NA A priori power analysis: NA	Initial dose: NA Target trough: NA PK sampling: Whole blood Was PK sampling at steady state?: NA Quantitation: HPLC/UV LOD/LOQ: NA	ISx regimen: SIR, MMF OR Azathioprine, GS (*n* = 64) OR SIR, Cyclosporine or TAC, GS^e^ (*n* = 36) Steroid dosing: NA	**Allele**	**Trough/(Dose/Weight)** (ng/mL) per (mg/kg) Mean ± SD	
				*1/*3 *n* = 5	294 ± 181	
				*3/*3 *n* = 50	349 ± 209	
Lee et al. (2014)	Clinical Trial N = 85 KT Self–reported race: Chinese – Han nationality Age: 42.9 ± 10.4 years Child: No Included: Stable KT treated with SIR for >3 months Excluded: pts taking drugs that precipitate DDI A priori power analysis: yes	Initial dose: NA Target trough: 5–10 ng/mL PK sampling: whole blood samples drawn 24 hours after the previous dose (before the next dose) Was PK sampling at steady state?: NA Quantitation: HPLC Detection: NA LOD/LOQ: NA	ISx regimen: SIR, MMF, Prednisone Steroid dosing: NA	**Allele**	**Trough/(Dose/Weight)** (ng/mL) per (mg/kg) Mean ± SD	
				*1/*1 *n* = 8 *1/*3 *n* = 36	200 ± 75.2	
				*3/*3 *n* = 41	290 ± 92.1	
Wang et al. (2014)	Open–label non–randomized clinical trial N = 24 KT Self–reported race: Chinese Age: 39.7 ± 11.1 years Child: No Included: at least 2 months after primary or secondary KT, stable sirolimus dose for >2 weeks Excluded: pregnant/nursing; prior or concurrent non–renal transplants; rejection in preceding 4 weeks, pts taking drugs that precipitate DDI with SIR or affect drug absorption A priori power analysis: NA	Initial dose: NA Target trough: NA PK sampling: trough: immediately before the sirolimus dose on days 1, 2, 3. AUC: 0.5, 1, 1.5, 2, 2.5, 3, 4, 5, 6, 8, 12, and 24 hours post-dose on day 3. Was PK sampling at steady state?: Yes Quantitation: LC–MS/MS LOQ: 0.25 ng/mL LOD: NA	ISx regimen: SIR + Prednisone (*n* = 23); MMF (*n* = 21); Cyclosporine (*n* = 9) Steroid dosing: NA	**Allele**	**Apparent Oral Clearance** (L/F/hour) Median (range)	
				*1/*1 *n* = 3	15.8 (12–22)	
				*1/*3 *n* = 8	10.9 (6–14)	
				*3/*3 *n* = 13	7.3 (3–16)	
Khaled et al. (2016)	Retrospective case series N = 173 HCT Self–reported race: 91 Caucasian, non–Hispanic, 52 Hispanic, 23 Asian/Pacific Islander, 7 other Age: 46 (10–70) years Child: No Included: Allogeneic HCT Excluded: 4 of the original 177 genotyped were excluded due to low-quality genotype sample A priori power analysis: NA	Initial dose: 12 mg (loading dose) on day –3, followed by 4 mg/day, with subsequent doses personalized to target levels Target trough: 3–12 ng/mL PK sampling: whole blood samples; time of sample collection relative to dose is NA; collected twice weekly for 100 days, reported results from first 14 days post–HCT Was PK sampling at steady state?: NA Quantitation: microparticle enzyme immunoassay LOD/LOQ: NA	ISx regimen: SIR + TAC ± Methotrexate Steroid dosing: not used	**Allele**	**Trough/Dose** (ng/mL) per (mg) Median (Range)	
				*1/*1 *n* = 8^f^	2.6 (1.8–8.9)	
				*1/*3 *n* = 40^f^	2.0 (0.6–5.6)	
				*3/*3 *n* = 121	2.1 (0.6–12.3)	
Li et al., (2015)	Clinical study N = 43 KT Self–reported race: Chinese Age: 35 (34–46) years Child: No Included: first KT, SIR for >1 month, stable post–KT without rejection Excluded: NA A priori power analysis: NA	Initial dose: 0.04 – 0.06 mg/kg/day Target trough: 5–10 ng/mL PK sampling: immediately before the next dose Was PK sampling at steady state?: NA Quantitation: automated enzyme immunoassay analyzer LOD/LOQ: NA	ISx regimen: SIR, MMF, Prednisolone Steroid dosing: Methylprednisolone 1000 mg IV at the time of KT, 500 mg IV next 2 days, followed by 80 mg/day oral Prednisone tapered to 10 mg/day to 20 mg until 3 months post–KT, reduced to 5 mg/day or discontinued	**Allele**	**Trough/(Dose/Weight)** (ng/mL) per (mg/kg/day) Median (Range)	
				*1/*1 *1/*3 *n* = 20	249 (248–410)	
				*3/*3 *n* = 23	389 (294–538)	
Rodriguez-Jimenez et al. (2017)	Retrospective cohort study N = 48 KT Self–reported race: NA Age: 58 ± 9 years Child: No Included: Age >18 years, received KT between 2002–2006, Excluded: pts taking drugs that precipitate DDI with SIR A priori power analysis: NA	Initial dose: NA Target trough: NA PK sampling: Whole blood samples; time of sample collection relative to dose is NA; collected at 1 week (W1), 2 weeks (W2), 1 month (M1), 3 months (M3), 6 months (M6)^g^ Was PK sampling at steady state?: Yes Quantitation: microparticle enzyme technique LOD/LOQ: NA	ISx regimen: SIR, MMF, Steroid Steroid dosing: NA	**Allele**	**Trough/(Dose/Weight)** (ng/mL) per (mg/kg) Mean ± SD (n)	
				*1/*1 *n* = 0 *1/*3 *n* = 8	W1	106 ± 29.3 (*n* = 3)
					W2	79.4 ± 45.9 (*n* = 3)
					W1+2	92.7 ± 37.5 (*n* = 6)
					M3	220 ± 85.9 (*n* = 2)
					M6	266 (*n* = 1)
				*3/*3 *n* = 39	W1	193 ± 133 (*n* = 33)
					W2	140 ± 65.5 (*n* = 25)
					W1+2	179 ± 116 (*n* = 39)
					M3	277 ± 236 (*n* =15)
					M6	233 ± 77.9 (*n* =13)
Zhang et al. (2017)	Clinical Trial N = 31 Healthy male volunteers Self–reported race: Chinese Age: 19 – 27 years Child: No Included: Body mass index 18–24 kg/m^2^, had stopped any other drug therapy for 2 weeks before study participation Excluded: History of drug allergies A priori power analysis: NA	Initial dose: 5 mg once Target trough: Not applicable PK sampling: before and 0.5, 1, 1.5, 2, 2.5, 3, 4, 6, 8, 12, 24, 48, 72, 96, 120, and 144 hour after dose Was PK sampling at steady state?: NA Quantitation: LC–MS/MS LOD: NA LOQ: 0.5 ng/mL	ISx regimen: Not applicable Steroid dosing: Not used	**Allele**	**AUC**_**0–144hr**_ (hr × ng/mL) Mean ± SD	
				*1/*1 *n* = 2	314 ± 129^h^	
				*1/*3 *n* = 14	440 ± 146	
				*3/*3 *n* = 15	550 ± 138	

Abbreviations: CNI, Calcineurin inhibitor; CYP, Cytochrome P450 Enzyme; DDI, Drug–Drug interaction with sirolimus; expressors, *1/*1 and *1/*3 genotypes which encode for CYP3A5 protein expression; F, fraction of sirolimus dose absorbed; HCT, Hematopoietic cell transplant; HPLC, high–performance liquid chromatography; hr, Hour; ISx, Immunosuppression; KT, Kidney transplant; LC–MS, Liquid Chromatography Mass Spectrometry; LLOQ, Lower limit of Quantitation; LOD, Limit of Detection; LOQ, Limit of Quantitation; MMF, Mycophenolate mofetil; CYP3A5 protein non-Expressors, *3/*3 genotype; NA, Not Available; PK, Pharmacokinetic; Pts, Patients; SIR, Sirolimus (rapamycin); SNP, Single nucleotide polymorphism; TAC, Tacrolimus; UV, Ultra–Violet spectroscopy.

^a^ Steroids affect CYP3A activity McCune et al. (2000) and sirolimus pharmacokinetics Cattaneo et al. (2004) and Mourad et al. (2005).

^b^ The number of participants in the SIR–based therapy group differed between 51 stated participants, but only 31 were purportedly included based on the description of CYP3A5 expressors (*n* = 18) and CYP3A5 non-expressors (*n* = 13).

^c^ The additional ISx administered to the sirolimus and tacrolimus was not stated; no statistically significant difference was observed in this sirolimus pharmacokinetic endpoint in the 24 participants receiving sirolimus and tacrolimus.

^d^ This publication stated there were no CYP3A5*1/*1 patients, but sirolimus pharmacokinetic data were reported for this genotype.

^e^ Assuming “GS” is an abbreviation for glucocorticoids, but this abbreviation was not defined.

^f^ This study is the only one that is sufficiently powered in this table based in a power analysis, published in 2015 (Emoto et al., 2015b), using pre-dose concentrations simulated with the PBPK model indicated that at least 80 participants in an enrichment design, 40 CYP3A5 expressers and 40 non-expressers, would be required to detect a significant difference in the predicted trough concentrations at 1 month of therapy (*p* < 0.05, 80% power).

^g^ Time–points after 6 months were not reported because participants were lost to follow–up, leading to an insufficient sample size for comparison.

^h^ Apparent oral clearance (L/hour) also differed based on *CYP3A5* genotype (*p* < 0.05).

Le Meur had a similar finding that non-expressors of CYP3A5 consistently had higher dose–adjusted trough concentrations and AUC_0–9hr_ when measured at 1 week, 2 weeks, 1 month, and 3 months post–KT (Le Meur et al., 2006). Miao et al. (2008), Lee et al. (2014), and Li et al. (2015) reported that the *CYP3A5* non-expressors have a higher trough concentration/(dose/weight) than expressors. These three studies had a more even distribution of expressors and non-expressor in their treatment groups (i.e., Miao *n* = 21,26; Lee *n* = 36, 41; Li = 20, 23) compared to other studies with more patients with the expressor genotype (Miao et al., 2008; Lee et al., 2014; Li et al., 2015). In a long–term retrospective cohort study, Rodriguez–Jimenez reported an association of CYP3A5 expressor status with the sirolimus concentration/dose ratio at weeks 1 and 2 post–KT values (Rodriguez-Jimenez et al., 2017).

In addition to *CYP3A5* rs776746, other *CYP3A5* SNPs (i.e., rs4646453 and rs15524) have been evaluated for their association with sirolimus pharmacokinetics (Tamashiro et al., 2017; Liu et al., 2021). Liu reported that rs776746 is in strong linkage disequilibrium with rs4646453 and rs15524 in 69 Chinese KT recipients. In rs4646453, homozygous AA alleles were associated with lower dose–adjusted trough concentrations when compared to homozygous CC (*p* < 0.001), with CC being the more dominant genotype (*n* = 41) in the study population (Liu et al., 2021). In rs4646453, homozygous GG (*n* = 6) had lower dose–adjusted trough concentrations compared to homozygous AA (*n* = 36) (*p* < 0.001). Tamashiro also evaluated rs15524 and only found a significant association between genotype and dose–adjusted trough concentration at 9 months post–KT (*p* < 0.05) (Tamashiro et al., 2017).

Over the past 20 years, there have been 14 studies of the *CYP3A5* rs776746 genotype to sirolimus pharmacokinetic phenotype studies in transplant patients. Table 2 summarizes the studies evaluating the *CYP3A5* rs776746 genotypes associated with sirolimus pharmacokinetics. Nine of the fourteen studies included in this review found a significant association between the sirolimus adjusted trough value or AUC and *CYP3A5* genotype (Anglicheau et al., 2005; Le Meur et al., 2006; Miao et al., 2008; Lee et al., 2014; Li et al., 2015; Rodriguez-Jimenez et al., 2017; Tamashiro et al., 2017; Zhang et al., 2017; Liu et al., 2021). The majority (80%) of these studies were conducted with less than 100 participants. An *a priori* power calculation could not be found in these studies, which leaves the question if statistically insignificant results were due to an underpowered sample size. Notably, a power analysis published in 2015, using pre-dose concentrations simulated with the PBPK model, indicated that at least 80 participants in an enrichment design, 40 CYP3A5 expressers, and 40 non-expressers, would be required to detect a significant difference in the predicted trough concentrations at 1 month of therapy (*p* < 0.05, 80% power) (Emoto et al., 2015b). Only one of the studies in Table 2 has 40 CYP3A5 expressors (Khaled et al., 2016). Furthermore, the studies were in heterogenous patient populations, with inconsistent eligibility criteria regarding drugs that precipitate a DDI with sirolimus. In addition, many studies had varying use of corticosteroids which affect CYP3A activity (McCune et al., 2000) and sirolimus pharmacokinetics (Cattaneo et al., 2004; Mourad et al., 2005). Thus, the studies were too heterogeneous and lacked adequately powered and sufficiently controlled studies for it to be feasible to establish a *CYP3A5* genotype to sirolimus pharmacokinetic phenotype association. Novel *CYP3A5* haplotypes are being identified and may yield insightful results (Rodriguez-Antona et al., 2022). Thus, we stress collaborative efforts to improve the accessibility of pharmacogenetic information to the entire pharmacogenetics community through the PharmGKB (Rodriguez-Antona et al., 2022). However, more research is needed regarding using preemptive *CYP3A5*-guided sirolimus for children.

### 5.2 Pharmacometabolomics

Metabolomics, which is the study of small molecule metabolite profiles in biological samples, is an additional promising new technology in precision medicine (Nicholson et al., 2002; Clayton et al., 2006; Clayton et al., 2009; Phapale et al., 2010; Wishart, 2019). Metabolomic experiments are occasionally categorized as targeted or untargeted (Wishart, 2019). The targeted metabolomic analysis involves evaluating a selected group of metabolites, often quantifying the metabolite concentrations relative to an authentic reference standard. In untargeted experiments, an unbiased approach is used, and all of the metabolites detected above the sensitivity threshold of the technology employed are analyzed. We demonstrated that pre–dose metabolomic profiling of plasma could predict busulfan clearance (Lin et al., 2016; Navarro et al., 2016; McCune et al., 2022b). Although the blood concentrations of many metabolites are tightly regulated (Homuth et al., 2012), we have found that the plasma metabolome does change after treatment with alkylating agents such as busulfan or cyclophosphamide (McCune et al., 2022a; McCune et al., 2022b).

The urinary metabolome is also of interest, as urine metabolite concentrations can vary widely and may serve as a “readout” of metabolic capacities that are not detected in blood (Schlosser et al., 2020). For tacrolimus, which is eliminated *via* similar drug-metabolizing enzymes and transporters as sirolimus, predose urine metabolites are associated with tacrolimus pharmacokinetics (Phapale et al., 2010). Further research is needed in this area, especially accounting for reduced kidney function resulting from concomitant cyclosporine or tacrolimus with sirolimus in transplant recipients. In 1,627 participants of the UK Biobank with reduced kidney function, the combination of metabolite quantitative trait loci revealed novel candidates for biotransformation and detoxification reactions (Schlosser et al., 2020). Thus, these novel biotransformation and detoxification reactions could influence the predose urinary metabolome in patients treated with sirolimus. Furthermore, the potential for renal metabolism of sirolimus should be considered because renal CYP3A can metabolize CYP3A-substrates (Dai et al., 2004; McCune et al., 2005).

## 6 Point-of-care sample collection of dried blood spots for precision dosing of sirolimus

Another approach to improving the precision dosing of sirolimus is simplifying the collection of the whole blood samples used for sirolimus TDM. There has been extensive interest in using dried blood spot (DBS) as a point-of-care method for obtaining blood samples to be used in sirolimus TDM. DBS sampling is a blood sampling method alternative to venipuncture and requires less blood volume, which makes it an attractive option for children. The DBS sampling process is not difficult to perform and does not require a trained phlebotomist for blood spot collection (Fokkema et al., 2009; Wagner et al., 2016). The patient provides a venipunctures capillary blood drop, places the blood onto a filter card, and subsequently allows the blood spot to dry. The DBS sample is then sent to a laboratory to quantify the sirolimus concentration with LC–MS (Wagner et al., 2016). DBS sampling may improve patient satisfaction by reducing commute times and possibly improving the precision dosing of sirolimus (Dickerson et al., 2015). For DBS to replace whole blood samples, it is important to evaluate if they provide similar sirolimus concentrations. Sirolimus concentration in DBS may be lower than in venous whole blood samples because drug concentrations tend to be lower in capillary blood (Klak et al., 2019). Veenhof et al. (2019) collected DBS and whole blood samples from patients and found that 76.9% of the samples were within acceptable limits.

The accurate quantitation of sirolimus concentration in DBS sampling depends on various factors, summarized in Supplementary Table S2. These factors range from 1. patient characteristics, 2. depositing the blood drop on the filter card; 3. the effect of how long it takes for the blood to dry on the filter card (i.e., drying time); 4. storing and transporting the DBS sample from the patient’s home to the laboratory; 5. punching the DBS section for quantitation; 6. extracting and quantitating sirolimus concentrations.

Table 3 summarizes the findings regarding the quantitation of sirolimus concentrations (abbreviated [SIR] in Table 3) from DBS samples. The patient’s characteristics, specifically their hematocrit and the sirolimus concentration at the time of the DBS collection, appreciably change the accuracy of sirolimus concentrations in DBS. Varying hematocrit influences the blood viscosity, the drying time needed for a DBS, and the potential interference with analyte recovery (De Kesel et al., 2013; Klak et al., 2019). Low hematocrit (less than 0.20 L/L) and high hematocrit (greater than 0.5 L/L) are associated with lower sirolimus recovery from DBS (den Burger et al., 2012; Koster et al., 2013). To obtain reliable sirolimus concentrations, it is optimal that the patient’s hematocrit range is between 0.23 and 0.50 L/L (den Burger et al., 2012; Koster et al., 2013; Koster et al., 2017). Therefore, DBS samples with high or low hematocrit concentrations must be corrected and interpreted cautiously (Klak et al., 2019). Also, better accuracy is achieved when sirolimus concentrations are at least 3.0 ng/mL; however, this may limit the use of DBS as trough concentrations may be lower than 3.0 ng/mL (Koster et al., 2015a; Koster et al., 2017).

**TABLE 3:** Studies to quantitate sirolimus concentrations ([SIR]) in dried blood spots prepared in the laboratory or obtained from patients taking sirolimus. Publications are organized in order of publication of the researcher group, starting with the group’s oldest publication.

References	Methods: DBS samples and [SIR] quantitation	Results and interpretation for [SIR]^a^
Den Burger et al., (2012) VU University Medical Center	DBS Sample Preparation • Lab staff performed all experiments using prepared DBS by combining purchased EDTA whole blood with plasma. • Hct average: 0.33 (range 0.22–0.41) • DBS volume: 20, 40, 60, 80, and 100 µL • [SIR]: Not clearly stated. Appear to be the low QC of [SIR] 1.57 ng/mL and the high QC of 23.5 ng/mL. Used an unknown volume of [SIR] 200,000 ng/mL^b^ to spike into the blood for subsequent experiments • Compared DBS to whole blood samples collected in EDTA	Hct Effect (1)
		• Low Hct levels were associated with poorer recovery outcomes, but the specific value for [SIR] was not reported.
		Blood Spot Volume (2.3)
		• Volume of DBS (40–100 µL) was within 85%–115% at two [SIR]
		• 20 µL DBS volume had a white edge of the unspotted paper which contributed to inaccuracy
		Punch Size and Location (5)
	[SIR] Quantitation using LC-MS • Calibration curves made with sirolimus–free EDTA whole blood (Hct: NA) • Matrix effect = −0.63% and were within desired limits • STD curve: 1.24, 2.24, 6.71, 11.2, 17.9, 35.8 ng/mL • LOQ: 1.12 ng/mL, which had an accuracy and precision of 80%–120% • LOD: NA • QCs were acceptable with accuracy and precision of 85%–115% over 6 replicates • *a priori* for acceptance: 85%–115% recovery	• Punch location did not influence accuracy. The peripheral punch/center punch ranges from 100.2% (low QC) to 109.9% (high QC)
Sadilkova et al., (2013) Seattle Children’s Hospital	DBS Sample Preparation • Patient samples: EDTA whole blood sample obtained from children (*n* = 68) taking SIR • Hct median level 30%–35% for all participants • Stability of [SIR] in patient’s samples (presumably whole blood) tested for 5 days at the following temperatures: 20°C, 25°C, 37°C, 60°C • DBS preparation by laboratory personnel • 50 µL blood on Whatman 903 DBS card • DBS volume: 25, 35, 50, 75, and 100 µL • Samples dried for 3 hr at room temperature. • Stability of [SIR] DBS for QC evaluated at −20°C, 4°C, 25°C	Overall Conclusion
		• [SIR] in DBS correlates with [SIR] in whole blood
		Hct (1)
		• No effect between Hct of 20%–45%
		Blood Spot Volume (2.3)
		• No effect.
		Stability of Analyte (4)
		• [SIR] degraded at 60°C within 24 hr.
	[SIR] Quantitation using LC-MS • Calibration curves made with immunosuppressant–free EDTA whole blood, with Hct of 30%–35%. • STD curve 1.2, 2.5, 5, 10, 20, and 40 ng/mL • LOQ and LOD: NA • Intra–run CV was 8.6% at 4 ng/mL and 5.9% at 20 ng/mL (*n* = 23 DBS) • Inter–run CVs were 14.8% at 4 ng/mL and 11.6% at 20 ng/mL (*n* = 25 DBS, stored at −20°C in–between quantitation, which occurred over 76 days) • *a priori* for acceptance: NA	Punch Size and Location (5)
		• No effect
Dickerson et al. (2015) Seattle Children’s Hospital	DBS Sample Preparation • Patient samples: A trained phlebotomist collected paired capillary DBS and venous blood samples. • 25 sample pairs (i.e., DBS and venous blood were obtained within minutes of each other from 34 children (median age: 13 years) who had received a solid organ transplant • [SIR] compared in three different types of samples: 1. Venous blood sent to the clinical lab for quantitation; 2. That same venous blood was used to create a whole blood spot (WBS) and stored until the DBS arrived; 3. A trained phlebotomist prepared the capillary DBS card. The DBS card was provided to the family to take home. Families were instructed to send the DBS card back to the hospital within 1 week. Sirolimus dose range: 0.4–4 mg twice daily [SIR] Quantitation using LC-MS, as described by Sadilkova et al. (2013). • *a priori* for acceptance: NA	Overall Conclusion
		• A small but statistically significant negative bias (0.6 ng/mL, *p* = 0.0011) was observed between the venous blood to the capillary DBS mailed back to the laboratory.
		• Analysis of [SIR] in DBS is possible, with the difference between venous and capillary blood within clinically acceptable limits.
		Extraction Recovery (6.2)
		• Comparing the venous whole blood to the DBS, the Bland–Altman analysis showed a difference in the [SIR] in these samples, with a larger variation at high [SIR]. In DBS, [SIR] were lower by a mean of 0.8 ng/mL (interquartile range = 1.9, *p* = 0.029)
		• Comparing the WBS to the DBS, there was no statistically significant difference
		• Comparing the venous blood to the WBS, [SIR] was lower by a mean of 1 ng/mL (*p* = 0.003) in WBS.
		• There are varying effects on drug concentrations with collecting capillary blood. Still, capillary draws often occur in clinical care, and the blood sample source (i.e., capillary vs. venipuncture) is not distinguished clinically.
	
Hempen et al. (2015) Spark Holland	DBS Sample Preparation • Lab staff prepared using purchased whole blood • Varying amounts of plasma were added or removed to achieve different target Hct values and [SIR]. • Hct: 0.25 or 0.60 • [SIR]: 1, 5, 50 ng/mL • Blood dried for at least 2 hr at room temperature.	Overall Conclusion
		• Temperature–enhanced desorption increased [SIR] recovery
		Extraction Recovery (6.2)
		• With FTD–LC–MS–MS, Hct did not impact the recovery of [SIR] from the DBS
	[SIR] Quantitation using temperature-enhanced flow–through desorption (FTD)–LC–MS–MS. FTD desorbs dried blood from the filter by perpendicular flushing solvent through the DBS in a chamber. The chamber’s inlet is connected to a solvent pump, and its outlet is connected to a collection device. Using the FTD removes the need to punch out the disc from a DBS. • STD curve: 0.2–100 ng/mL • LOQ and LOD: NA • Bias and QCs: NA • Within–run CV: 2.6%–6.3% • *a priori* criteria for acceptance: not explicitly stated, but appears to be within 15%	
Koster et al. (2013)	DBS Sample Preparation • Lab staff performed all experiments using prepared DBS by combining packed red blood cells with pooled immunosuppressant–free human serum. Placed 50 µL of blood on the DBS card and dried at room temperature for 24 h. An 8 mm disc from the central part of the DBS was punched, extracted, and [SIR] quantitated. • Evaluated Hct of 0.20, 0.25, 0.30, 0.35, 0.40, 0.45, and 0.50 L/L at 3, 20, 40, 50 ng/mL [SIR] • Evaluated 31 ET CHR paper and Whatman FTA DMPK–C DBS over the eight [SIR] concentrations below • Standardized 0.35 L/L DBS were prepared at low and high concentrations with volumes of 30, 50, 70, and 90 µL (50 µL was the reference for bias calculations) • Stability in DBS evaluated on − autosampler 10°C for 5 days − at 22°C and 37°C for multiple times points over (*N* = 5 replicates) • Compared these DBS to whole blood samples[SIR]: 1, 3, 10, 20, 25, 30, 40, and 50 ng/mL [SIR] Quantitation using LC-MS • STD curve 1, 3, 10, 20, 25, 30, 40, 50 ng/mL • LOQ: 1 ng/mL • LOD: NA • *a priori* criteria for acceptance: Bias and QCs with 15% were acceptable (number of replicates was NA)	Overall Conclusion
Groningen		• Highest overall bias 13.0% for 1 ng/mL
		• Protein binding in the blood and hydrogen binding to the cellulose of the paper may influence extraction
		Overall comparison of DBS to whole blood
		• *R* ^2^ > 0.871 between the DBS and whole venous blood
		• Passing–Bablok regression^c^ showed <12% slope
		Hct Effect (1) • Significantly influenced results, with improved bias when the measured concentrations were corrected for the effect of Hct Choice of Filter Card (2.1) • No significant difference between 31 ET CHR paper and Whatman FTA DMPK–C DBS cards Blood Spot Volume (2.3) • Volume of DBS (i.e., 30, 50, 70, and 90 µL) had a minor effect on [SIR]. Stability of DBS (4) • Stable on autosampler at 10°C for 5 days • Stable at 22°C for 7 days • Degraded at 37°C over 4-week Extraction Recovery (6.2) • After correction, most biases were within the acceptable range of 15% bias • Extraction recovery was highest at low [SIR] and high Hct concentrations; lowest at high [SIR] and low Hct − Highest extraction recovery of 93% at [SIR] 3 ng/mL and an Hct of 0.45 L/L − Lowest extraction recovery of 69% at [SIR] 50 ng/mL and an Hct of 0.25 L/L − −20% bias at [SIR] 40 ng/mL and Hct 0.20 L/L
Koster et al. (2015a)	DBS Sample Preparation • Lab staff performed all experiments using purchased whole blood. Varying amounts of plasma were added or removed to achieve different Hct values and [SIR]. Placed 50 µL of blood on the DBS card and dried at room temperature for 24 h. An 8 mm disc from the central part of the DBS was punched, extracted, and [SIR] quantitated Koster et al. (2013) • Hct: 0.10, 0.20, 0.30, 0.40, 0.50, & 0.60 L/L	Overall Conclusion
Groningen		• Extraction recovery depended on [SIR] and Hct in all DBS cards
		• The DBS cards performed differently, particularly at extreme [SIR] and Hct
	• [SIR]: 3 or 100 ng/mL • Tested the following DBS cards: − Whatman 31 ET CHR − Whatman FTA DMPK–C − Whatman 903 − Perkin Elmer 226 − Agilent Bond Elut DMS	[SIR] Effect (1)
		• At [SIR] 3.0 ng/mL, bias exceeded 15% with an Hct of 0.1 L/L, and bias was acceptable with an Hct of 0.2–0.6 L/L • At [SIR] 100 ng/mL, bias exceeded 15% with an Hct of 0.1 or 0.2 L/L, and bias was acceptable with an Hct of 0.3–0.6 L/L
		Hct Effect (1)
		• Hct had a minor effect at [SIR] 3 ng/mL
		Choice of Filter Card (2.1)
		• Whatman DMPK–C cards had the highest extraction recoveries and most consistent performance.
		• Advised not to use different DBS card types in routine clinical analysis and to fully (re)validate the analytical method if the filter card is changed.
	[SIR] Quantitation using LC-MS • STD curve range, LOQ, LOD, CV: NA. LC–MS method not specifically referenced, but is presumably the previously reported method Koster et al. (2013). • *a priori* criteria for acceptance: Bias and QCs with 15% were acceptable (number of replicates was NA)	
Koster et al. (2015b)Groningen	DBS Sample Preparation • Lab staff performed all experiments using DBS prepared using purchased citrate whole blood. Varying amounts of plasma were added to achieve different Hct. Placed 50 µL of blood on the DBS card and dried at room temperature. An 8 mm disc from the central part of the DBS was punched, extracted, and [SIR] quantitated Koster et al. (2013). • Hct: 0.1, 0.2, 0.3, 0.4, 0.5, 0.6 L/L • [SIR]: 100 ng/mL • DBS Card: Whatman FTA DMPK–C without chemicals • Dried over 3, 24, or 48 hr at ambient temperature	Overall Conclusion
		• Advised a drying time of at least 24 hr to stabilize Hct and [SIR] recovery in DBS.
		• Different drying times can introduce significant bias in [SIR] between patient DBS samples and the laboratory-prepared standards used for LC-MS quantitation.
		Effect of Drying Time (3)
		• The extraction recovery of [SIR] changed based on Hct. The extraction recovery was significantly different between 3 and 24 h, with the recovery
		• At Hct of 0.1 L/L, the extraction recovery of [SIR] was higher at 3 compared to 24 h and was stable from 24 to 48 hr.
	[SIR] Quantitation using LC–MS. The LC–MS method is not specifically referenced but presumably the previously reported method Koster et al. (2013). • *a priori* criteria for acceptance: NA	• At Hct of 0.4 L/L, the extraction recovery of [SIR] was lower at 3 compared to 24 h and was stable from 24 to 48 hr.
		• The CV for all [SIR] and Hct combinations was within 10%.
Koster et al. (2017)	DBS Sample Preparation^d^ • Lab staff performed all experiments using DBS prepared using purchased citrate whole blood. Varying amounts of plasma were added to achieve different Hct. Placed varying volumes of blood on one type of DBS card (i.e., Whatman FTA DMPK–C without chemicals) and dried at room temperature. An 8 mm disc from the central part of the DBS was punched, extracted, and [SIR] quantitated Koster et al. (2013).• Hct: 0.23, 0.28, 0.33, 0.38, 0.43, 0.48, and 0.53 L/L (0.38 L/L was the reference for bias calculations) • [SIR]: 3 or 100 ng/mL • Blood spot volume: 30, 50, and 70 µL (50 µL was the reference for bias calculations)	Overall Conclusion
		• Hct did not affect [SIR] as much at these lower and more clinically relevant [SIR] of 3 and 10 ng/mL, a slightly higher Hct range (0.23–0.53 L/L) and a better performing DBS card (i.e., Whatman DMPK-C).
		Hct Effect (1)
		• Bias and CVs were acceptable for Hct of 0.23, 0.33, 0.43, 0.48, and 0.53 L/L. Bias was acceptable for Hct of 0.28 L/L at [SIR] 10 ng/mL but not acceptable at [SIR] 3 ng/mL.
	[SIR] Quantitation using LC–MS described in Koster et al. (2013). At LOQ for [SIR] of 1 ng/mL, the overall CV of 14.7% and an overall bias of −0.9. • *a priori* criteria for acceptance: Bias and CVs with 15% were acceptable	Blood Spot Volume (2.3)
		• Bias and CVs were acceptable for DBS volumes of 30 and 70 µL compared to 50 µL.
Veenhof et al. (2019) Groningen	DBS Sample Preparation • Compared paired patient whole blood and DBS samples were collected during routine visits to the hospital using a home sampling technique available online. • Patient samples: A trained phlebotomist collected paired DBS and whole blood samples. 39 sample pairs (i.e., DBS–whole blood sample) were obtained within 10 min of each other from 56 adults receiving sirolimus for solid organ or hematopoietic cell transplantation. • Hct 0.4 (range: 0.23–0.51) v/v • [SIR]^e^ –in whole blood 5.0 ± 2.4 (range: 1.9–10.9) ng/mL –in DBS 4.7 ± 1.9 (range: 1.8–9.7) ng/mL • Two drops of blood onto Whatman FTA DMPK–C DBS card, allowed to dry for 24–74 h at room temperature, packed in a zip lock mini bag with a desiccant, and then transported to the laboratory. DBS samples are stored at −20°C until quantitation for up to 29 weeks (the maximum length of time that the DBS are stable).	Overall Conclusion
		• Passing–Bablok regression showed no significant constant or systematic difference.^d^
		• Bland–Altman showed the mean ratio of whole blood to DBS is 1.0 (95% confidence interval of 0.93–1.07).
		Only one of the two *a priori* limits of acceptance was met.
		1. Over 67% of the paired samples, specifically 76.9%, were within ±20% of the mean of both methods, meeting this criterion.
		2. Less than 80%, specifically 77.3%, of the samples were within the range of 85%–115% around the ratio of the paired samples, not meeting this criterion [CLSI; Zwart et al. (2018)]. Because this criterion was not met, DBS sampling cannot replace whole-blood sampling at this time.
		Hct Effect (1)
		• Hct did not appear to influence DBS results
	[SIR] Quantitation over the standard curve range of [SIR] 1–50 ng/mL, using the LC–MS method described in Koster et al. (2013), Koster et al., 2015a, and Koster et al. (2015b). *a priori* criteria of acceptance: 1. at least 67% of the paired samples should be ±20% of the mean of both methods 2. at least 80% of the samples should be within the range of 85%–115% around the ratio of the paired samples	

Abbreviations: AUC, Area under the plasma concentration–time curve; CV, Coefficient of variation; DBS, Dried blood spot; FTD, Flow–through desorption; Hct, Hematocrit; LC–MS, Liquid chromatography–mass spectrometry; LOD, Limit of detection; LOQ, Limit of quantitation; NA, Not available; ND, Not detectable; QC, Quality control; [SIR], Sirolimus concentration; STD, Standard deviation; WBS, Whole blood sample.

^a^ Organized using categories of Supplementary Table S2.

^b^ Original publication stated 0.2 mg/mL; we converted to ng/mL to be consistent with other manuscripts summarized in this table.

^c^ A Bland–Altman test is a mathematical analysis that compares similar variables that may have utilized different techniques to evaluate the same variable. Passing–Bablok regression analysis compares two different mechanisms or techniques that calculate an acceptable bias Giavarina (2015).

^d^ Paired patient whole blood and DBS samples were collected during routine visits to the hospital using a home sampling technique available online. Unfortunately, not enough paired samples were collected in patients taking sirolimus so data was not presented.

^e^ Shown as mean + standard deviation (range).

The second factor influencing sirolimus concentrations from DBS is depositing the blood spot on the filter card. Hydrogen bridges are known to form between sirolimus and cellulose filters (Klak et al., 2019). Because the sirolimus concentration is influenced by the type of filter card used, it is recommended that a laboratory use only one type of filter card for calibration purposes (Koster et al., 2015a). The Whatman FTA DMPK–C, 31 ET CHR, and Whatman DMPK–C filter cards have consistent sirolimus extraction recovery (Koster et al., 2013; Koster et al., 2015a). However, the Whatman 903 and Ahlstrom 226 filter cards are preferred because of their untreated cellulose filter and because they comply with CLSI guidelines (Klak et al., 2019; CLSI document EP09-A3, 2013). In addition to the effects of the filter card, the blood spot volume and homogeneity of its placement within one spot affect the accuracy of sirolimus concentrations from DBS samples. Filter card oversaturation by blood spot volumes equal to or greater than 100 µL (den Burger et al., 2012; Sadilkova et al., 2013) and volumes less than 20 or 30 µL can lead to inaccurate results (den Burger et al., 2012; Koster et al., 2013; Koster et al., 2017). Patients are more likely to provide low blood spot volumes when self–sampling than the recommended 50 µL blood spot volumes (Klak et al., 2019). Dickerson and others evaluated point-of-care (i.e., at-home) collection by providing and educating families about how to collect and mail DBS samples back to the laboratory (Dickerson et al., 2015). A small negative, but not statistically significant, bias between DBS and whole blood samples was found. The sirolimus concentrations in the DBS samples were within clinically acceptable limits (Dickerson et al., 2015). Although this is encouraging for self-sampling, additional studies in children are needed to evaluate if they can provide the recommended blood spot volume studied to date (den Burger et al., 2012; Koster et al., 2013; Koster et al., 2017; Klak et al., 2019; Veenhof et al., 2019).

The third and fourth factors influencing sirolimus concentrations are drying the filter card and storing and transporting the DBS on the filter card, respectively. DBS samples are recommended to be dried at room temperature and away from light for at least 24 hr to allow for accurate hematocrit effects during sirolimus recovery (Koster et al., 2015b; Klak et al., 2019). DBS samples can be stored for 20–29 weeks in the lab at −20°C before losing the stability of sirolimus concentrations (Koster et al., 2015b; Veenhof et al., 2019). All DBS samples require spot-checking for appropriate volume size (Veenhof et al., 2019). Sirolimus DBS samples degrade rapidly within 24 hr at 60°C, but sirolimus is relatively stable at 25°C (Sadilkova et al., 2013; Klak et al., 2019). Therefore, patients using DBS sampling must be educated on properly collecting, storing, and transporting their DBS samples.

The fifth and sixth factors influencing DBS’ sirolimus concentrations are punching out the DBS section for quantitation and the subsequent extraction and quantitation of the sirolimus concentrations, respectively. Punch size and location of the DBS do not appear to influence sirolimus concentrations (den Burger et al., 2012; Sadilkova et al., 2013; Koster et al., 2017; Klak et al., 2019). The highest extraction and recovery rates of sirolimus were found at high hematocrit and low sirolimus concentrations (Table 3) (Koster et al., 2013). When utilizing flow-through desorption (FTD) with LC-MS-enhanced temperature desorption, sirolimus recovery improved significantly (Hempen et al., 2015). However, further trials are still needed to confirm whether FTD–LC–MS–MS can be established as an accurate DBS tool.

In summary, some factors (i.e., hematocrit, sirolimus concentration, filter card, drying time) influence sirolimus concentrations from DBS. However, patient education is necessary for parents to collect sufficient blood spot volumes at the correct time (Dickerson et al., 2015; Klak et al., 2019). In addition, potentially losing samples in the mail is an ongoing concern (Dickerson et al., 2015; Urquhart and Knauer, 2015). Therefore, precision dosing of sirolimus using DBS samples is not recommended for children.

## 7 Point-of-care collection of saliva or sweat for precision dosing of sirolimus

Other matrices, such as saliva or sweat, can also be used in TDM. As a common alternative to a blood sample, the non-invasive and easily accessible nature of saliva samples makes it optimal for TDM in outpatient settings and potentially in children. Furthermore, with the help of PBPK modeling, the system drug exposure may be predicted from those non-plasma samples. Using PBPK models, we hypothesize that sirolimus concentrations in saliva or sweat can be related to whole blood concentrations to predict sirolimus exposure.

### 7.1 Precision sirolimus dosing based on salivary concentrations

Compared to collecting whole blood samples, collecting saliva samples is non-invasive, which can allow for more frequent sample collection and facilitate at–home personalization of sirolimus doses. Multiple factors, including saliva collection time, method, and the device, should all be carefully considered for sample collection. Generally, devices made of polyester and polyethylene outperform cotton in quantitating some (but not all) proteins, steroids, and small molecules (e.g., antidepressants, theophylline, and caffeine) (Ghareeb and Akhlaghi, 2015). A summary of the saliva collection methods and devices used in clinical trials and studies involving children is provided (Table 4). For younger children who cannot voluntarily spit or chew, uniquely designed saliva collection devices may be needed to aid sample collection. For TDM, unstimulated saliva samples were collected by expectorating or with the help of a saliva collection device such as Salivette^®^ (Sarstedt, Nümbrecht, Germany). In some cases, saliva excretion is stimulated by paraffin wax (Cohen et al., 1985; Tarantino et al., 2018) or citric acid (Kirk et al., 1994; Kopecky et al., 1997) and collected by similar methods. For younger children, saliva collection is usually performed with the help of an adult and a variety of devices and collection methods to facilitate sample collection.

**TABLE 4:** Studies of saliva collection in children.

Age^a^	Device used	Volume collected^b^	Analytes/tests	References
Newborn	• Vacuum aspiration from the floor of the mouth into a plastic silicon–coated tube	200 µL	Caffeine	Lee et al. (1996)
	• Collection time: <10 min			
Neonates	• Infant’s Swab	NA	Gentamycin	Samb et al. (2022)
	• Collection time: 90 s			
3–10 days	• Infant swab	>120 µL	Oxytocin	Vittner et al. (2018)
	• Collection time: 5–10 min			
6–7 days old with very low birth weight	• Portable suction with low pressure (<100 mmHg), saliva was collected using 1–mL sterile plastic syringes with a blunt end attached to the suction tube.	1,000–2000 µL	Testosterone; Cortisol	Cho et al. (2021)
	• Collection time: 5–10 min			
From birth–20 weeks old	• SalivaBio Infant Swab	200–1,000 µL	Leptin; adiponectin	Linares et al. (2021)
	• Collection time: 60–90 s or until the lower third of the swab was saturated.			
1–9 months	• Salimetrics infant swab	NA	Interleukin–1β; interleukin–1 receptor antagonist; immunoglobulin A	Miller (2021)
	• Collection time: NA			
≤18 months	• Sorbette	NA	Cotinine	Wang et al. (2018)
	• Collection time: NA			
2–30 months	• Pacifier–based collection device or SalivaBio’s Children’s Swab	158 μL with swab	NA	Novak (2021)
	• Collection time: NA	174 μL with pacifier		
3–6 months of age	• Salivette swab	>300 μL	Proteomics; Amylase activity	Morzel et al. (2011)
	• Collection time: 2 min maximum.			
4–6 months	• Chew Swab and mopping up pooled saliva in mouth and face (the kind of swab not specified)	NA	Secretory immunoglobulin A	Xiao et al. (2017)
	• Collection time: 30–60 s			
Approximately 6 months	• Sponge Oragene™ DNA self-collection kits (OG-250)	NA	DNA methylation	Moccia et al. (2021)
	• Collection time: NA			
<6 years	• Wipe the oral cavity with the Salivette swab or chew on Salivette	NA	17–hydroxyprogesterone	Neumann et al. (2021)
	• Collection time: 3 min			
1.3–19 years	• Modified medical pacifiers or Salivette swabs	NA	Busulfan	Rauh et al. (2006)
	• Collection time: 2 min			
>2 years	• Suction with a disposable plastic pipette or expectorate into a plastic collection container	250 µL unstimulated or citric acid-stimulated saliva when patients do not have sufficient saliva	Lamotrigine	Ryan et al. (2003)
	• Collection time: NA			
Pediatric patients, age not specified	• Mucous extractor or a syringe or expectorate saliva directly into labeled specimen containers.	1,000–2000 μL saliva by citric acid stimulation	Morphine	Kopecky et al. (1997)
	• Collection time: NA			

Abbreviations: min, minutes; NA, Not available.

^a^ Using the American Medical Association definition of ages (AMA, 2020): Neonates or newborns: birth to 1 month; Infants: 1 month to 1 year; Children: 1 through 12 years; Adolescents: 13 through 17 years (may also be referred to as teenagers depending on the context); Adults: 18 years or older; Older adults: 65 years and older).

^b^ Salivary collection was unstimulated unless stated otherwise.

Most drugs enter the saliva by passive diffusion, and the salivary concentration is potentially proportional to its free and non-ionized fraction in the blood (Haeckel, 1993). A drug’s partition into saliva largely depends on the physiochemical properties of the drug, including protein binding, lipophilicity, ionization, molecular weight, and other confounding factors such as saliva flow rate, pH, and composition (Ghareeb and Akhlaghi, 2015). For example, the saliva to the plasma concentration ratio of drugs such as digoxin and primidone fluctuates significantly with the salivary flow rate. However, phenytoin and carbamazepine are less influenced by flow rate, likely due to their high lipophilicity (Haeckel, 1993). Sirolimus is highly lipophilic, as indicated by its high logP value (Supplementary Table S1); thus, its saliva concentration is predicted to be less affected by salivary flow rate.

Other factors, such as saliva pH and active saliva–secreting glands, may also affect the saliva-to-plasma ratio of drugs. Saliva stimulation increases the saliva production from the parotid gland, which may increase the salivary concentration of some drugs, such as diazepine, that are present higher in the parotid saliva (DiGregorio et al., 1978). Saliva pH can vary due to physiological factors and saliva flow rate (Tremblay et al., 2012). The actual pH determines the ionized portion of a drug in saliva; drugs that have pKa of less than 5.5 and greater than 8.5 exist in saliva (pH 5.8–7.8) in their non-ionized form. Sirolimus has a pKa value of 10.40 ± 0.07 (Supplementary Table S1), thus, should be affected less by changes in salivary pH (Haeckel, 1993; Ghareeb and Akhlaghi, 2015).

Compared to blood sampling, saliva sampling is non-invasive, which allows for more frequent collection and self-sampling (Ghareeb and Akhlaghi, 2015). Sirolimus was detected in the saliva of 4 adults with stable sirolimus trough concentration (Nudelman et al., 2013). However, in addition to the limited sample size, there was substantive variability in the ratio of sirolimus blood to saliva concentrations (range: not detected to 6.68). Therefore, more data is needed to determine if and how saliva could be used for pharmacokinetic sampling to enable precision dosing of sirolimus.

### 7.2 Precision sirolimus dosing using sweat concentrations

Sweat samples are used to evaluate if a patient has taken a drug of abuse (Chawarski et al., 2007; Barnes et al., 2009). Innovations in biosensing technology have enabled *in situ* sweat analysis (Tai et al., 2018; Wang et al., 2022), which has led to an interest in using sweat as a potential matrix for point-of-care precision dosing of sirolimus. Eccrine sweat glands are the most abundant sweat glands in most body surface areas. They contain a secretory coil and a duct (Baker and Wolfe, 2020). Sweat is secreted by cells on the secretory duct, and drug excretion into sweat mainly *via* passive diffusion. Research showed that the secretion of drugs into sweat depends on the free form of the drug in the plasma, the partition coefficient, and the pKa of the drug (Johnson and Maibach, 1971).

Several additional factors must be considered when considering sweat as a matrix for sirolimus TDM. 1. Is sirolimus excreted in sweat? 2. Is sirolimus metabolized or transported by the eccrine glands? 3. Is sirolimus metabolized by the skin, which may occur during sweat collection? 4. Does sweat gland density or sweat collection vary with anatomic regions (for example, can we collect sweat from the wrist using a wearable such as FitBit?). CYP3A4 is the most active human form of CYP for sirolimus metabolism, while PgP is the most active transporter for sirolimus. CYP3A4, 3A5, and 3A7 mRNA have been detected in the skin. However, their expression levels are lower than in the liver (Baron et al., 2008), and CYP3A activity in the skin is weak (Kazem et al., 2019). PgP is also expressed in dermal components, including sweat ducts, vessels, nerve sheaths, and muscles of human skin, and a moderate expression in the basal epidermis (Skazik et al., 2011). The presence of CYP3A in the skin and PgP expression on sweat ducts suggest that sirolimus could be metabolized or transported during sweat collection. These factors should be carefully considered while relating the drug concentration in sweat to that in the blood.

Other factors, such as variations in sweat rate and sweat gland density between individuals and anatomical regions, should be counted to establish a method for TDM using sweat. Sweat gland density varies among different skin surfaces. The average sweat gland densities on the finger, palm, back of the hand, wrist, forearm, forehead, and back of the neck are 441, 318, 290, 212, 171, 208, and 110 glands/cm^2^, respectively (Bariya et al., 2020). The dorsal hand, forehead, and upper back are among the regions that have the highest sweat flow during passive heating (Taylor and Machado-Moreira, 2013). Thus, the hand region could be a good target for sweat collection. In addition, there is individual variation in sweat perspiration rate (Sato and Sato, 1983). Those factors should be considered when optimizing methods for sweat collection.

A commonly used method for sweat collection is absorptive sweat patches, which can be left on the skin for a prolonged period (up to 3 weeks). Then, the absorbed sweat content is tested for illegal drugs [e.g., cocaine (Burns and Baselt, 1995) and fentanyl (Schneider et al., 2008)]. In this situation, the drug quantitation in sweat is limited to a “yes, the drug is present” or “no, the drug is not present” question. The concentration of the substance could not be quantitatively related to the intake amount. Thus, this method is not suitable for precision dosing of sirolimus. Another useful method for accessing sweat is iontophoresis sweat induction which has been widely used in diagnosing cystic fibrosis in children and adults (Accurso et al., 2014). An FDA-approved iontophoresis device, such as Macroduct^®^, has been used for iontophoretic sweat induction. It passes a tiny current (0.5 mA) through the skin to deliver the cholinergic agonists to stimulate the sweat gland locally to accelerate perspiration. Sweat is collected within 30 min after induction. This iontophoresis method enables on-demand sweat access which is optimal for TDM where continuous analytes monitoring may be beneficial (Bandodkar and Wang, 2014).

The muscarinic agonist, pilocarpine, was initially used for sweat collection to diagnose cystic fibrosis testing. It was reported to produce a localized sweating effect for up to 90 min (Simmers et al., 2018a). Carbachol has recently been used for longer durations of sweat collection. Carbachol is a synthetic choline ester that is a parasympathomimetic that mimics the effect of acetylcholine on both the muscarinic and nicotinic receptors. Carbachol is slowly metabolized by cholinesterase and may have a longer-lasting sweat-stimulating effect than pilocarpine (Simmers et al., 2018a). With carbachol induction (17.5–568 µg, Table 5), sweat is detected in the directly stimulated regions for over 5 hr (Sonner et al., 2017). We have also found in an ongoing trial that carbachol-based sweat stimulation could achieve more stable and effective sweat collection in a clinical setting.

**TABLE 5:** Carbachol dose for sweat induction by iontophoresis.

Age (year)^a^	Device^b^	Electrical current	Total charge	References
			Theoretical carbachol dose^c^	
NA	Wescor Nanoduct	0.5 mA for 2 min	74 mC^d^	Simmers et al. (2018b)
			142 μg	
NA	Device Designed by authors	0.2 mA for 2.5 min	30 mC	Sonner et al. (2017)
			57 μg	
NA	Wescor Nanoduct	0.5 mA for 18.75–150 s	9.24–73.92 mC	Simmers et al. (2018a)
			17.5–140.0 ug	
30.3 ± 12.3	NA	1 mA for 5 min	300 mC	Braune et al. (2001)
			568 μg	
50.0 ± 12.3	NA	1 mA for 5 min	300 mC	Birklein et al. (1997)
			568 μg	
NA	Custom–designed wearable system	0.1 mA for 5 min	30 mC	Wang et al. (2022)
			57 μg	

Abbreviations: min, minutes; NA, Not available.

^a^ Reported as mean ± standard deviation (number under 18 years of age is not reported).

^b^ All studies used 1% Carbachol for iontophoresis.

^c^ The theoretical carbachol dose is calculated by $\frac{Total\;charge}{Faraday\;constant\;of\;96,485\;C/mol}\times \frac{183\;g}{mol}$ where 96,485 C/mol is the Faraday constant and 183 g/mol is the molecular weight of carbachol (National Library Of Medicine, 2023); the actual delivered carbachol by iontophoresis may be less than 30% of the theoretical carbachol dose Simmers et al. (2018a).

^d^ This publication uses a different method to estimate the total charge. Specifically, it includes the additional charge delivered during the starting and ending of the iontophoresis current. Two additional iontophoresis sessions were administered within 24 hr.

More recently, carbachol has been used as a cholinergic agent to stimulate sweating with the newer sweat collection technology (Tai et al., 2018; He et al., 2019; Yang et al., 2020) to achieve real-time on-body sweat collection. Recently, a wearable sensor patch design incorporating a carbachol iontophoresis module can achieve on-demand sweat collection and real-time drug or metabolite quantitation (Wang et al., 2022). This technology could potentially revolutionize the field of precision dosing if successfully applied to this field. However, we could not find publications using carbachol for sweat induction in children (Table 5). To evaluate the safety of carbachol use, we summarized the literature on carbachol use in children (Supplementary Table S3). Carbachol, at doses less than 1.2 mg for inhalation, has been administered to evaluate bronchial response in children. This dose is higher than the carbachol dose delivered by iontophoresis (17.5–568 µg, Table 4) for sweat collection. Thus, carbachol could be safely used for sweat collection in children, although such studies have yet to be conducted. Because of the potential benefit of a point-of-care monitoring of sweat, such studies will hopefully be conducted.

## 8 Ongoing pilot studies

In combination with tacrolimus, sirolimus can be used to prevent GVHD in recipients of an allogeneic hematopoietic cell transplant (HCT) (Khaled et al., 2016; McCune and Bemer, 2016; McCune et al., 2016). Because GVHD persists as a significant source of non-relapse mortality (Bidgoli et al., 2022), we are describing preliminary data from two pilot studies seeking to improve the dosing of sirolimus and tacrolimus in HCT recipients.

### 8.1 Feasibility of preemptive *CYP3A5*–guided dosing

For tacrolimus, the CPIC summarized the published literature supporting the association of the *CYP3A5* genotype with dose–adjusted trough concentrations of tacrolimus (Birdwell et al., 2015). CPIC also provided dosing recommendations for tacrolimus based on known *CYP3A5* genotypes, facilitating the use of this pharmacogenetic test for patient care (Birdwell et al., 2015). Thus, an increasing number of solid organ transplant and HCT programs are implementing preemptive *CYP3A5*–guided tacrolimus dosing (Van Driest et al., 2014; Woillard et al., 2017). Tacrolimus and sirolimus have similar dispositions, both being metabolized by CYP3A4 and CYP3A5 and transported by PgP. However, CPIC does not provide dosing recommendations for sirolimus based on the *CYP3A5* genotype. That is not surprising with the lack of compelling data on a *CYP3A5* genotype to sirolimus pharmacokinetic phenotype association in heterogeneous populations with various pharmacokinetic endpoints (Table 2).

To take our first step towards the “right–dose–first–time” paradigm (Minto and Schnider, 1998) with tacrolimus, we conducted a pilot study to determine if CYP3A5*3 results can be obtained before the first tacrolimus dose is administered in 30 allogeneic HCT recipients. Thirty participants were enrolled between January and July 2022; all signed informed consent approved by the Institutional Review Board (COH Protocol 21233) of the City of Hope before study procedures started. Unfortunately, due to a delay at the genotyping laboratory (ARUP Laboratories, Salt Lake City, UT), the CYP3A5*3 results were not promptly obtained in one participant. However, that participant was one of four participants who did not receive an allogeneic HCT and, thus, did not receive tacrolimus or sirolimus. Of those 26 participants, their age was 64.5 (median, range: 26–79) years, and 13 were men. They were receiving their first allogeneic HCT to treat acute myeloid leukemia (*n* = 14; 53.9%), myelodysplastic syndrome (*n* = 7, 26.9%), and acute lymphocytic leukemia (*n* = 5, 19.2%). The majority were conditioned with non-myeloablative conditioning (*n* = 20, 76.9%) before a peripheral blood progenitor cell (*n* = 26, 100%) transplant from an HLA-mismatched unrelated, HLA identical sibling, Haploidentical or HLA-matched related donor, respectively (46.2%, 19.2%, 19.2%, 15.4%, respectively). The prescribed GVHD prophylaxis with tacrolimus and sirolimus (±other) is 61.5%, post-transplant cyclophosphamide with mycophenolate mofetil with either tacrolimus or sirolimus is 23.1%, and various other regimens (15.3%).

Of the 26 participants undergoing an allogeneic HCT, 24 received tacrolimus, and 21 received sirolimus. In general, precision dosing of tacrolimus and sirolimus doses was as follows: tacrolimus trough concentrations targeted between 5 and 15 ng/mL, and sirolimus trough concentrations targeted between 5 and 10 ng/mL (Khaled et al., 2016). The interpatient variability in the initial trough concentration divided by the initial dose divided by total body weight [(ng/mL) per (mg/kg)] by CYP3A5*3 genotype is shown in Table 6. However, it should be noted that the purpose of this research is not to evaluate these genotype–phenotype relationships but to work towards implementing *CYP3A5*–guided tacrolimus dosing. The initial trough concentration was evaluated before dose adjustments to achieve the target trough concentration. Some initial trough concentrations were obtained before the third dose, so they were not likely to be at steady-state. However, because of the frequency of dose adjustments and the long half-lives of both drugs, it was difficult to ascertain if steady-state was ever achieved. These pilot results support harmonization and standardization for the pharmacokinetic modeling and dose recommendations of these immunosuppressants and immunosuppressant assays, the latter of which was recently suggested (Veenhof et al., 2019). Genotyping was performed at an offsite laboratory, ARUP Laboratories (Salt Lake City, UT, United States). Patients were genotyped for SNPs in the CYP3A5*3 genes using Polymerase Chain Reaction/Fluorescence Monitoring for CYP3A5*3 rs776746, c.219-237A>G (ARUP Laboratories, 2022). These assays’ reference SNP ID number was CYP3A5*1>*3 (rs776746). A minority [7 of 24 (29.2%) tacrolimus, 3 of 19 (15.7%) sirolimus] participants had at least one copy of the CYP3A5*1 allele, which agrees with Hardy–Weinberg equilibrium for those who self-identify as Caucasian. The *CYP3A5* genotype results were available 25 (3–175) days before the first tacrolimus dose. Thus, further supporting that preemptive *CYP3A5*-guided dosing is feasible even when the genotyping laboratory is off-site.

**TABLE 6:** Interpatient variability in the Trough per Dose/Weight [(ng/mL) per (mg/kg)] shown by CYP3A5*3 genotype^a^.

CYP3A5 *3 genotype	*n*	Tacrolimus trough	*n*	Sirolimus trough
*1/*1 and *1/*3	7	428.0 (233–1,125)	3	74.0 (52.3–130)
*3/*3	17	391.7 (204–649)	16	92.0 (18.9–251)

^a^ Data is shown as n: number of participants and median (range) of the first trough concentration (ng/mL) obtained per the initial dose (mg) divided by total body weight (kg). Total body weight is the actual body weight immediately before HCT. First trough concentrations were not consistently obtained at steady-state.

^b^ Descriptive statistics are shown only; no statistical analysis for the *CYP3A5* genotype to pharmacokinetic phenotype will be conducted because it was not part of this pilot study in a small and heterogeneous patient population.

### 8.2 Feasibility of wearables to quantitate sirolimus

Although DBS does not appear feasible for sirolimus (Section 6), the recent development of wearables (Wang et al., 2022) may allow for point-of-care monitoring of sirolimus concentrations, which opens the opportunity for adherence monitoring and model MIPD at home. Toward that goal, we are conducting a pilot study to determine the feasibility of wearables to quantitate drug concentrations in plasma, saliva, and sweat in cancer patients (Clinicaltrials, 2022; NIH). Three allogeneic HCT recipients taking sirolimus had plasma, saliva, and sweat samples collected. None of the iontophoresis with pilocarpine yielded sufficient sweat volume to quantitate sirolimus. Thus, sweat collection using carbachol should be pursued (Table 5). In addition, the carbachol dose used for iontophoresis is lower than that used previously in children (Supplementary Table S3). Thus, we suggest future studies use carbachol for sweat induction in children and adults. The sirolimus concentrations ranged from 0.135 to 0.243 ng/mL in plasma and from 0.55 to 0.79 ng/mL in saliva, which may be detected using a wearable.

## 9 Conclusion

Dosing sirolimus based on trough concentrations is the current standard of care, although MIPD will be beneficial (McCune and Bemer, 2016; McCune et al., 2016). Therefore, the use of MIPD should be implemented. To date, the data do not suggest the use of DBS for point-of-care monitoring of sirolimus concentrations for precision dosing of sirolimus. Future research should focus on the use of preemptive *CYP3A5*–guided sirolimus dosing or the use of wearables for point-of-care quantitation of sirolimus in blood, saliva, or sweat.
