# Supplementary material for: Precision sirolimus dosing in children: The potential for model-informed dosing and novel drug monitoring
Source: Front Pharmacol. 2023 Mar 20;14:1126981. doi: 10.3389/fphar.2023.1126981 (PMC10069443; doi:10.3389/fphar.2023.1126981)
Supplement: Supplementary file 1 [file DataSheet1.docx]

Supplemental Methods: Pubmed searches and PRISMA diagrams

Search Methods for Section 5.1. and Table 2. (see Supplemental Figure 1 for PRISMA diagram)

Literature searches were conducted on PubMed using the search terms "sirolimus AND CYP3A5 polymorphisms" and "("Sirolimus"[Mesh]) AND "CYP3A5 protein, human" [Supplementary Concept]. Combining these searches resulted in 81 records, from which 48 manuscripts were removed from the initial title and abstract review. No automated screening was used in this search. 33 manuscripts were assessed for eligibility, leading to the removal of an additional 19 publications. Publications were removed for not reporting the desired PK values in *CYP3A5* genotypes (n=7) or not reporting original data (n=12). Therefore, 14 publications were included in this review.

Search Methods for Section 6 and Table 3 (see Supplemental Figure 2 for PRISMA diagram).

Literature searches were conducted on PubMed using the search terms ("Sirolimus"[Mesh]) AND "Dried Blood Spot Testing"[Mesh]). The search resulted in 13 records, from which 3 publications were removed from the initial title and abstract review. After further screening, 10 relevant publications were identified. Next, 10 records were retrieved from the relevant publications. Finally, 3 more reports were identified via cross reference. Therefore, 13 publications were included in the literature review discussion, but only 9 were included in this table.

Search Methods for Section 7.1 and Table 4 (see Supplemental Figure 3 for PRISMA diagram).

Literature searches were conducted on PubMed using the search terms "Saliva AND therapeutic drug monitoring" filter=clinical trial and randomized clinical trial. The search resulted in 99 records, from which 19 publications were removed from the initial title and abstract review. After further screening, 14 relevant publications are identified. 5 records were retrieved from the 14 relevant publications. 10 more publications were identified via cross reference. Therefore, 15 publications were included in this table.

Search Methods for Section 7.2 and Table 5 (see Supplemental Figure 4 for PRISMA diagram).

Literature searches were conducted on PubMed using the search terms "carbachol AND iontophoresis." The search resulted in 100 records, from which 89 publications were removed from the initial title and abstract review. 4 relevant publications were further identified and retrieved. 1 more article was identified by cross reference. Overall, 5 publications were included in this review.

Search Methods for Supplemental Table 3 (see Supplemental Figure 5 for PRISMA diagram).

Literature searches were conducted on PubMed using the search terms "Carbachol" filter=human and children. The search resulted in 204 records. After abstract review, 197 publications were removed, 7 relevant records were identified and retrieved. Overall, 7 publications were included in this table.

**Identification of studies via databases and registers (Table 2)**

Records removed *before screening*:

Duplicate records removed (n = 14)

Unable to access (n=4)

Records removed for other reasons (n = 30)

Records identified from:

Pubmed (n = 81)

**Identification**

Records excluded (n=0)

Records screened

(n = 33)

Reports sought for retrieval

(n = 33)

Reports not retrieved (n = 0)

**Screening**

Reports assessed for eligibility

(n = 33)

Reports excluded:

Did not report Sirolimus PK parameters for CYP3A5 Genotypes (n = 7)

Reviews (n = 10)

Book Chapter (n = 1)

Simulation only (n=1)

Studies included in review

(n = 14)

**Included**

**Supplemental Figure 1. Search methods for pharmacogenomics (Section 5.1 and Table 2)**.

**Identification of studies via other methods**

**Identification of studies via databases and registers (Table 3)**

Records identified from:

Websites (n =0 )

Organisations (n = 0 )

Citation searching (n =3)

Records removed *before screening*:

Duplicate records removed (n = 0)

Records marked as ineligible by automation tools (n = 0)

Non-relevant records removed (n = 0)

Records identified from Pubmed:

Databases (n = 13)

Registers (n = 0 )

**Identification**

Records excluded (n = 3)

Records screened

(n =13)

Reports not retrieved (n = 0)

Reports assessed for eligibility

(n = 3)

Reports sought for retrieval

(n = 3)

Reports sought for retrieval

(n = 10)

Reports not retrieved (n = 0)

**Screening**

Reports excluded: (n =0)

Reports excluded (n = 0)

Reports assessed for eligibility

(n = 10)

Studies included in review

(n = 13)

**Included**

**Supplemental Figure 2. PRISM diagram of search strategy dried blood spot sampling for sirolimus (Section 6, Table 3)**

**Identification of studies via other methods**

**Identification of studies via databases and registers (Table 4)**

Records identified from:

Websites (n =0 )

Organisations (n = 0 )

Citation searching (n =10)

Records removed *before screening*:

Duplicate records removed (n = 0)

Records marked as ineligible by automation tools (n = 0)

Non-relevant records removed (n = 19)

Records identified from Pubmed:

Databases (n = 99)

Registers (n = 0 )

**Identification**

Records screened

(n =80)

Records excluded (n = 66)

Reports not retrieved (n = 0)

Reports sought for retrieval

(n = 10)

Reports sought for retrieval

(n = 14)

Reports not retrieved (n = 9)

**Screening**

Reports assessed for eligibility

(n = 10)

Reports excluded (n = 0)

tc.

Reports excluded: (n = 0)

Reports assessed for eligibility

(n = 5)

Studies included in review

(n = 15)

**Included**

**Supplemental Figure 3. PRISM diagram of search strategy for saliva collection in children (Section 7.1 & Table 4)**

**Identification of studies via other methods**

**Identification of studies via databases and registers (Table 5)**

Records identified from:

Websites (n =0 )

Organisations (n = 0 )

Citation searching (n =1 )

Records removed *before screening*:

Duplicate records removed (n = 0)

Records marked as ineligible by automation tools (n = 0)

Records removed for other reasons (n = 89)

Records identified from Pubmed:

Databases (n = 100)

Registers (n = 0 )

**Identification**

Records screened

(n =12)

Records excluded (n = 7)

Reports not retrieved (n = 0)

Reports sought for retrieval

(n = 1)

Reports assessed for eligibility

(n = 1)

Reports sought for retrieval

(n = 5)

Reports not retrieved (n = 0)

**Screening**

Reports excluded (n=0)

Reports excluded (n =0)

Reports assessed for eligibility

(n = 5)

Studies included in review

(n = 6)

**Included**

**Supplemental Figure 4. Prism diagram for search strategy for carbachol use in sweat collection (Section 7.2, Table 5)**

**Identification of studies via databases and registers (Supplemental Table3)**

Records removed *before screening*:

Duplicate records removed (n = 0)

Records marked as ineligible by automation tools (n = 0)

Records removed for other reasons (n = 0)

Records identified from Pubmed:

Databases (n = 204)

Registers (n = 0)

**Identification**

Records screened

(n =204)

Records excluded

(n = 197)

Reports sought for retrieval

(n = 7)

Reports not retrieved (n = 0)

**Screening**

Reports excluded (n =0)

Reports assessed for eligibility

(n = 7)

Studies included in review

(n = 7)

**Included**

**Supplemental Figure 5. PRISM diagram for search strategy for carbachol use in children (Supplemental Table 3)**

**Supplemental Methods. Sirolimus quantitation in plasma, saliva, and sweat (Section 8.2).**

LC/MS grade methanol, formic acid (FA) and ammonium acetate were purchased from Fisher Scientific (Fair Lawn, NJ, USA). Water was purified using a Millipore IQ 7000 (Milford, MA, USA). Sirolimus was purchased from LC Laboratories (Woburn, MA, USA). Sirolimus–D3 was purchased from Cerilliant (Round Rock, TX, USA) and used as an internal standard (IS). Sample analysis was performed on an Acquity UPLC I–Class System interfaced with a Xevo TQ–XS Triple Quadrupole Mass Spectrometer (Waters, Milford, MA, USA). MassLynx version 4.2 software was used to acquire and analyze data.

To measure plasma, saliva and sweat concentrations of sirolimus over the range of 0.1–100 ng/mL, 10X standard spiking solutions were prepared at 1–1000 ng/mL in 70% methanol. 10X control spiking solutions were prepared at 5, 50 and 500 ng/mL. The internal standard (IS, sirolimus–D3) was prepared at a concentration of 0.5 ng/mL in methanol. 20 µl standard or control spiking solution (or 70% methanol blank for sample) was added to 200 µl of blank matrix (or sample) in a 1.7 mL micro centrifuge tube. The tube was vortex mixed and centrifuged briefly. 600 µl of methanol containing IS was added to precipitate proteins. The tube was vortex mixed for 60 seconds then centrifuged for 8 minutes at 4°C. A 50 µl aliquot of supernatant was diluted with 150 µl 2 mM ammonium acetate, 0.1% FA in 70% methanol. 10 µl was injected per sample.

Analyte separation was achieved on a Synergi 4μ Hydro–RP 75 x 2.00 mm analytical column (Phenomenex, Torrance, CA USA). The auto–injector temperature was maintained at 8^o^C and the column temperature at 50^o^C. A methanol gradient was used to elute the analytes from the column at a flow rate of 0.3 mL/min. Initial conditions of 50% solvent A (2 mM ammonium acetate, 0.1% FA in water) and 50% solvent B (2 mM ammonium acetate, 0.1% FA in methanol) was maintained for 0.5 minute. The concentration of solvent B was increased to 100% over 2 minutes and maintained for 4 minutes. Solvent B was returned to 50% and the column equilibrated for 3.5 minutes. Total run time was 10 minutes. The retention time was 3.22 minutes for sirolimus and sirolimus–D3.

The electrospray ionization source of the mass spectrometer was operated in positive ion mode with a cone gas flow of 150 L/hr and a desolvation gas flow of 1000 L/hr. The capillary voltage was set to 3.5 kV. The cone voltage was optimized at 38 V for sirolimus and 30 V for sirolimus–D3. The collision cell energy was 16 eV for both. The source temperature was 150^o^C and the desolvation temperature was 450^o^C. To minimize the mobile phase flow to the source, a solvent delay program was used from 0 to 1.8 minutes and from 5.2 to 10 minutes. The precursor→product ion combinations at m/z 931.84→864.92 for sirolimus and 934.84→864.93 for sirolimus–D3 were used in multiple reaction monitoring (MRM) mode to determinate these compounds. The use of MRM provided sufficient specificity and sensitivity. The lower limit of quantitation was 0.1 ng/mL.

| **Supplemental Table 1.** Clinical use and relevant physiochemical properties of sirolimus | | |
| --- | --- | --- |
| **Items** | **Clinical use and properties** | **Reference** |
| Current Dosing | Adult loading dose: 6–12 mg/day  Pediatric loading dose: 2.5 mg/m^2^/day | (Claxton et al., 2005; Alyea et al., 2008; Ho et al., 2009; Nakamura et al., 2012; Khaled et al., 2013; McCune et al., 2016) |
| Patient populations where trough or AUCs are associated with clinical outcomes | Solid organ and hematopoietic cell transplant  Autoimmune lymphoproliferative syndrome Lymphangioleiomyomatosis | (McCormack et al., 2011; Bride et al., 2016; McCune and Bemer, 2016; McCune et al., 2016) |
| Concentrations  (ng/mL) | Whole blood trough: 8.0 ± 4.6 (range, 1.8–21.6) | (Goyal et al., 2013; Nudelman et al., 2013) |
|  | Saliva: ND – 1.54^a^ | (Nudelman et al., 2013) |
|  | Sweat: Not available |  |
| Physical and chemical property | Molecular weight: 914.2  LogP: 4.3–4.63  pKa: 10.40 ± 0.70 | Data from Pubchem (Pubchem) and Scifinder^n^(Scifinder) |
| Plasma half–life | 62 + 16 hour | (McCune et al., 2016) |
| Protein Binding | Red Blood Cells | (McCune et al., 2016) |
| Metabolizing enzymes | Cytochrome (CYP) 3A4, 3A5, 2C8 | (Anglicheau et al., 2005; McCune et al., 2016) |
| Transporter | Efflux transport by p–glycoprotein | (Anglicheau et al., 2005; Glotzbecker et al., 2012; McCune et al., 2016) |
| Abbreviations: AUC, Area under the plasma concentration–time curve; ND, Not detectable | | |
| ^a^Blood concentrations at the time of saliva collection ranged from 3.79 – 6.75 ng/mL, dose of sirolimus not reported | | |

| **Supplemental Table 2.** Steps involved with dried blood spots collection and literature for quantitating sirolimus concentrations in dried blood spots (DBS) | | | |
| --- | --- | --- | --- |
| **Step**  **(in chronological order)** | | **Sub–steps** | **Publications evaluating how this step contributes to the variability in DBS** |
| 1 | Patient characteristics | 1 Hematocrit Effect | (den Burger et al., 2012; Koster et al., 2013; Koster et al., 2015a; Koster et al., 2017; Klak et al., 2019; Veenhof et al., 2019) |
| 2 | Depositing the blood drop on the filter card | 2.1 Choice of Filter Card  2.2 Blood Spot Homogeneity  2.3 Blood Spot Volume  2.4 Quality of Self–sampling | (den Burger et al., 2012; Koster et al., 2013; Sadilkova et al., 2013; Koster et al., 2015a; Koster et al., 2017; Klak et al., 2019; Veenhof et al., 2019) |
| 3 | Amount of time it takes for the blood to dry on the filter card | 3 Effect of Drying Time | (Koster et al., 2013; Koster et al., 2015b; Klak et al., 2019; Veenhof et al., 2019) |
| 4 | Storing & transporting the DBS from the patient’s home to the laboratory | 4 Stability of Analyte | (Sadilkova et al., 2013; Koster et al., 2015a; Veenhof et al., 2019) |
| 5 | Punching the DBS section for quantitation | 5 Punch Size and Location | (den Burger et al., 2012; Sadilkova et al., 2013; Dickerson et al., 2015; Klak et al., 2019; Veenhof et al., 2019) |
| 6 | Extracting and quantitating SIR concentrations | 6.1 Spot to Carry Over  6.2 Extraction Recovery  6.3 Matrix Effects | (Koster et al., 2013; Hempen et al., 2015; Veenhof et al., 2019) |
| Modified from Klak et al. (Klak et al., 2019) | | | |

| **Supplemental Table 3.** Carbachol use in children. | | |
| --- | --- | --- |
| **Population (Age Range)** | **Dose of inhaled carbachol**^a^ | **Reference**^a^ |
| Healthy (9 year) | 2,570 ± 700 ug | (Zapletal et al., 1991) |
| Healthy (3–6 year)  Asthma (3–6 year) | 400 ug in healthy children  83.1± 7.8 ug in children with asthma | (Badier et al., 1999) |
| Healthy (8–16 year)  Asthma (5–15 year) | Doses increased up to 1,200 ug or when 185% increase in respiratory resistance was reached. The provocative dose for a 50% increase in respiratory resistance was:  -656.8 ± 292.7 ug for healthy children  -376.5 ± 265.2 ug for children with asthma | (Sommer et al., 1993) |
| Healthy (11.8 + 2.3 year)  Asthma (8.7 + 2.6 year) | 240 – 1200 ug | (Kraemer et al., 1993) |
| Healthy (7–14 year)  Asthma (5–15 year) | The provocative dose was not reported. Carbachol administered up to 1000 ug or when 185% increase in respiratory resistance was reached | (Frey and Kraemer, 1997) |
| Footnotes: ^a^Carbachol was used for bronchial hyperreactivity testing; toxicity to carbachol was not reported. ^b^Two studies were not included because they did not state the number of children (less than 18 years) administered carbachol for transplanted submandibular glands secretion in patients between 17–51 years (Liu et al., 2016) or 15–44 years (Orehek et al., 1976). | | |

**References**

Alyea, E.P., Li, S., Kim, H.T., Cutler, C., Ho, V., Soiffer, R.J., et al. (2008). Sirolimus, tacrolimus, and low-dose methotrexate as graft-versus-host disease prophylaxis in related and unrelated donor reduced-intensity conditioning allogeneic peripheral blood stem cell transplantation. *Biol Blood Marrow Transplant* 14(8)**,** 920-926. doi: 10.1016/j.bbmt.2008.05.024.

Anglicheau, D., Le Corre, D., Lechaton, S., Laurent-Puig, P., Kreis, H., Beaune, P., et al. (2005). Consequences of genetic polymorphisms for sirolimus requirements after renal transplant in patients on primary sirolimus therapy. *Am J Transplant* 5(3)**,** 595-603. doi: 10.1111/j.1600-6143.2005.00745.x.

Badier, M., Guillot, C., and Dubus, J.C. (1999). Bronchial challenge with carbachol in 3-6-year-old children: body plethysmography assessments. *Pediatr Pulmonol* 27(2)**,** 117-123. doi: 10.1002/(sici)1099-0496(199902)27:2<117::aid-ppul8>3.0.co;2-h.

Bride, K.L., Vincent, T., Smith-Whitley, K., Lambert, M.P., Bleesing, J.J., Seif, A.E., et al. (2016). Sirolimus is effective in relapsed/refractory autoimmune cytopenias: results of a prospective multi-institutional trial. *Blood* 127(1)**,** 17-28. doi: 10.1182/blood-2015-07-657981.

Claxton, D.F., Ehmann, C., and Rybka, W. (2005). Control of advanced and refractory acute myelogenous leukaemia with sirolimus-based non-myeloablative allogeneic stem cell transplantation. *Br J Haematol* 130(2)**,** 256-264. doi: 10.1111/j.1365-2141.2005.05600.x.

den Burger, J.C., Wilhelm, A.J., Chahbouni, A., Vos, R.M., Sinjewel, A., and Swart, E.L. (2012). Analysis of cyclosporin A, tacrolimus, sirolimus, and everolimus in dried blood spot samples using liquid chromatography tandem mass spectrometry. *Anal Bioanal Chem* 404(6-7)**,** 1803-1811. doi: 10.1007/s00216-012-6317-8.

Dickerson, J.A., Sinkey, M., Jacot, K., Stack, J., Sadilkova, K., Law, Y.M., et al. (2015). Tacrolimus and sirolimus in capillary dried blood spots allows for remote monitoring. *Pediatr Transplant* 19(1)**,** 101-106. doi: 10.1111/petr.12392.

Frey, U., and Kraemer, R. (1997). Oscillatory pressure transients after flow interruption during bronchial challenge test in children. *Eur Respir J* 10(1)**,** 75-81. doi: 10.1183/09031936.97.10010075.

Glotzbecker, B., Duncan, C., Alyea, E., 3rd, Campbell, B., and Soiffer, R. (2012). Important drug interactions in hematopoietic stem cell transplantation: what every physician should know. *Biol Blood Marrow Transplant* 18(7)**,** 989-1006. doi: 10.1016/j.bbmt.2011.11.029.

Goyal, R.K., Han, K., Wall, D.A., Pulsipher, M.A., Bunin, N., Grupp, S.A., et al. (2013). Sirolimus pharmacokinetics in early postmyeloablative pediatric blood and marrow transplantation. *Biol Blood Marrow Transplant* 19(4)**,** 569-575. doi: 10.1016/j.bbmt.2012.12.015.

Hempen, C.M., Maarten Koster, E.H., and Ooms, J.A. (2015). Hematocrit-independent recovery of immunosuppressants from DBS using heated flow-through desorption. *Bioanalysis* 7(16)**,** 2019-2029. doi: 10.4155/bio.15.97.

Ho, V.T., Aldridge, J., Kim, H.T., Cutler, C., Koreth, J., Armand, P., et al. (2009). Comparison of Tacrolimus and Sirolimus (Tac/Sir) versus Tacrolimus, Sirolimus, and mini-methotrexate (Tac/Sir/MTX) as acute graft-versus-host disease prophylaxis after reduced-intensity conditioning allogeneic peripheral blood stem cell transplantation. *Biol Blood Marrow Transplant* 15(7)**,** 844-850. doi: 10.1016/j.bbmt.2009.03.017.

Khaled, S.K., Palmer, J., Stiller, T., Senitzer, D., Maegawa, R., Rodriguez, R., et al. (2013). A phase II study of sirolimus, tacrolimus and rabbit anti-thymocyte globulin as GVHD prophylaxis after unrelated-donor PBSC transplant. *Bone Marrow Transplant* 48(2)**,** 278-283. doi: 10.1038/bmt.2012.175.

Klak, A., Pauwels, S., and Vermeersch, P. (2019). Preanalytical considerations in therapeutic drug monitoring of immunosuppressants with dried blood spots. *Diagnosis (Berl)* 6(1)**,** 57-68. doi: 10.1515/dx-2018-0034.

Koster, R.A., Alffenaar, J.W., Greijdanus, B., and Uges, D.R. (2013). Fast LC-MS/MS analysis of tacrolimus, sirolimus, everolimus and cyclosporin A in dried blood spots and the influence of the hematocrit and immunosuppressant concentration on recovery. *Talanta* 115**,** 47-54. doi: 10.1016/j.talanta.2013.04.027.

Koster, R.A., Botma, R., Greijdanus, B., Uges, D.R., Kosterink, J.G., Touw, D.J., et al. (2015a). The performance of five different dried blood spot cards for the analysis of six immunosuppressants. *Bioanalysis* 7(10)**,** 1225-1235. doi: 10.4155/bio.15.63.

Koster, R.A., Botma, R., Greijdanus, B., Uges, D.R.A., Kosterink, J.G.W., Alffenaar, J.-W.C., et al. (2015b). The influence of the dried blood spot drying time on the recoveries of six immunosuppressants. *Journal of Applied Bioanalysis* 1(4)**,** 116-122. doi: 10.17145/jab.15.019.

Koster, R.A., Veenhof, H., Botma, R., Hoekstra, A.T., Berger, S.P., Bakker, S.J., et al. (2017). Dried blood spot validation of five immunosuppressants, without hematocrit correction, on two LC-MS/MS systems. *Bioanalysis* 9(7)**,** 553-563. doi: 10.4155/bio-2016-0296.

Kraemer, R., Sommer, C.W., Gschwend-Eigenmann, S., Schoni, M.H., and Stadler, B.M. (1993). Interfering factors to sensitivity and specificity of bronchial reactivity in children. *Pediatr Allergy Immunol* 4(4)**,** 187-195. doi: 10.1111/j.1399-3038.1993.tb00090.x.

Liu, X.J., Li, M., Su, J.Z., Wang, Z., Xie, Z., and Yu, G.Y. (2016). Carbachol improves the secretion of transplanted submandibular glands during the latent period after microvascular autologous transplantation for severe keratoconjunctivitis sicca. *Int J Oral Maxillofac Surg* 45(10)**,** 1273-1279. doi: 10.1016/j.ijom.2016.03.015.

McCormack, F.X., Inoue, Y., Moss, J., Singer, L.G., Strange, C., Nakata, K., et al. (2011). Efficacy and safety of sirolimus in lymphangioleiomyomatosis. *N Engl J Med* 364(17)**,** 1595-1606. doi: 10.1056/NEJMoa1100391.

McCune, J.S., and Bemer, M.J. (2016). Pharmacokinetics, Pharmacodynamics and Pharmacogenomics of Immunosuppressants in Allogeneic Haematopoietic Cell Transplantation: Part I. *Clin Pharmacokinet* 55(5)**,** 525-550. doi: 10.1007/s40262-015-0339-2.

McCune, J.S., Bemer, M.J., and Long-Boyle, J. (2016). Pharmacokinetics, Pharmacodynamics, and Pharmacogenomics of Immunosuppressants in Allogeneic Hematopoietic Cell Transplantation: Part II. *Clin Pharmacokinet* 55(5)**,** 551-593. doi: 10.1007/s40262-015-0340-9.

Nakamura, R., Palmer, J.M., O'Donnell, M.R., Stiller, T., Thomas, S.H., Chao, J., et al. (2012). Reduced intensity allogeneic hematopoietic stem cell transplantation for MDS using tacrolimus/sirolimus-based GVHD prophylaxis. *Leuk Res* 36(9)**,** 1152-1156. doi: 10.1016/j.leukres.2012.04.022.

Nudelman, Z., Keshet, N., Elhalal, M.D., Friedman, M., and Czerninski, R. (2013). Levels of Sirolimus in saliva vs. blood--the rationale of topical oral use for oral malignancy. *Anticancer Res* 33(2)**,** 661-663. doi: https://doi.org/10.1111/odi.12229

Orehek, J., Massari, J.P., Gayrard, P., Grimaud, C., and Charpin, J. (1976). Effect of short-term, low-level nitrogen dioxide exposure on bronchial sensitivity of asthmatic patients. *J Clin Invest* 57(2)**,** 301-307. doi: 10.1172/JCI108281.

Pubchem [*https://pubchem.ncbi.nlm.nih.gov/compound/Sirolimus*](https://pubchem.ncbi.nlm.nih.gov/compound/Sirolimus) [Online]. [Accessed Oct 13 2022].

Sadilkova, K., Busby, B., Dickerson, J.A., Rutledge, J.C., and Jack, R.M. (2013). Clinical validation and implementation of a multiplexed immunosuppressant assay in dried blood spots by LC-MS/MS. *Clin Chim Acta* 421**,** 152-156. doi: 10.1016/j.cca.2013.02.009.

Scifinder [*https://scifinder-n.cas.org/searchDetail/substance/634e2ebf3c1f076117f05c97/substanceDetails*](https://scifinder-n.cas.org/searchDetail/substance/634e2ebf3c1f076117f05c97/substanceDetails) [Online]. [Accessed Oct 17 2022].

Sommer, C.W., Frey, U., Schonli, M.H., and Kraemer, R. (1993). Specific approach on dose-response curves to inhaled carbachol assessed by the interruption technique in children. *Pediatr Res* 34(4)**,** 478-484. doi: 10.1203/00006450-199310000-00018.

Veenhof, H., Koster, R.A., Alffenaar, J.C., van den Berg, A.P., de Groot, M.R., Verschuuren, E.A.M., et al. (2019). Clinical application of a dried blood spot assay for sirolimus and everolimus in transplant patients. *Clin Chem Lab Med* 57(12)**,** 1854-1862. doi: 10.1515/cclm-2019-0053.

Zapletal, A., Sukova, B., and Pohanka, V. (1991). [Bronchial hyperreactivity in pediatric and adolescent asthmatics and healthy children]. *Cesk Pediatr* 46(10-11)**,** 449-458.
